# Priority effects shape the structure of infant-type *Bifidobacterium* communities on human milk oligosaccharides

**DOI:** 10.1038/s41396-022-01270-3

**Published:** 2022-06-29

**Authors:** Miriam N. Ojima, Lin Jiang, Aleksandr A. Arzamasov, Keisuke Yoshida, Toshitaka Odamaki, Jinzhong Xiao, Aruto Nakajima, Motomitsu Kitaoka, Junko Hirose, Tadasu Urashima, Toshihiko Katoh, Aina Gotoh, Douwe van Sinderen, Dmitry A. Rodionov, Andrei L. Osterman, Mikiyasu Sakanaka, Takane Katayama

**Affiliations:** 1 Graduate School of Biostudies, Kyoto University, Kyoto, Japan; 2 School of Biological Sciences, Georgia Institute of Technology, Atlanta, GA USA; 3 Sanford Burnham Prebys Medical Discovery Institute, La Jolla, CA USA; 4 Next Generation Science Institute, Morinaga Milk Industry Co., Ltd., Kanagawa, Japan; 5 Faculty of Agriculture, Niigata University, Niigata, Japan; 6 School of Human Cultures, The University of Shiga Prefecture, Hikone, Shiga Japan; 7 Department of Food and Life Science, Obihiro University of Agriculture and Veterinary Medicine, Obihiro, Hokkaido Japan; 8 APC Microbiome Ireland and School of Microbiology, Food Science Building, University College Cork, Cork, Ireland; 9 Present Address: Department of Food and Nutrition, Kyoto Women’s University, Kyoto, Japan

**Keywords:** Community ecology, Microbial ecology, Microbiome

## Abstract

Bifidobacteria are among the first colonizers of the infant gut, and human milk oligosaccharides (HMOs) in breastmilk are instrumental for the formation of a bifidobacteria-rich microbiota. However, little is known about the assembly of bifidobacterial communities. Here, by applying assembly theory to a community of four representative infant-gut associated *Bifidobacterium* species that employ varied strategies for HMO consumption, we show that arrival order and sugar consumption phenotypes significantly affected community formation. *Bifidobacterium bifidum* and *Bifidobacterium longum* subsp. *infantis*, two avid HMO consumers, dominate through inhibitory priority effects. On the other hand, *Bifidobacterium breve*, a species with limited HMO-utilization ability, can benefit from facilitative priority effects and dominates by utilizing fucose, an HMO degradant not utilized by the other bifidobacterial species. Analysis of publicly available breastfed infant faecal metagenome data showed that the observed trends for *B. breve* were consistent with our in vitro data, suggesting that priority effects may have contributed to its dominance. Our study highlights the importance and history dependency of initial community assembly and its implications for the maturation trajectory of the infant gut microbiota.

## Introduction

Aberrant development of the human gut microbiota during infancy is linked to various health conditions that can manifest themselves throughout adulthood [1]. However, little is known about how the microbial community, following its introduction to the gut, becomes established and structured. Past studies have shown that the postpartum microbial colonization period is critical for gut microbiota development, being influenced by various factors, such as delivery mode (vaginal vs caesarean section) and feeding regime (formula vs breastmilk) [2, 3]. The role played by deterministic factors such as host genetics, which may select for specific taxa through niche-based mechanisms, has previously been examined [4]. However, the impact of certain processes such as assembly history and priority effects, which are stochastic in terms of species colonization order and identity [5], on the structuring of host-associated gut microbial communities has not been fully addressed. Priority effects, i.e., the effect of arrival order of a strain/species in a particular environmental niche on species interactions and community composition [6], present an important mechanism that can lead to the formation of multiple community states, including ecologically important transient stable states [7]. Although the role of species colonization history in structuring the human gut microbiota is increasingly appreciated [8, 9], we know little about whether and how priority effects contribute to the assembly of bifidobacterial communities in the infant gut.

Bifidobacteria are among the first colonizers of the infant gut and commonly represent over 70% of the total gut microbial community during breastfeeding [10–12]. The early colonization by bifidobacteria is at least in part facilitated by their ability to metabolize human milk oligosaccharides (HMOs). HMOs are a group of complex unconjugated glycans that, despite being the third most abundant solid component in breastmilk, provide no (direct) nutritional value to the host as they are resistant to digestion by pancreatic enzymes [13]. Instead, HMOs act as highly selective agents that promote the growth of certain bifidobacteria [14–17], though HMO-utilization strategies differ significantly by species and strain. Furthermore, while breastfeeding has been associated with an overall higher prevalence and abundance of bifidobacteria [18, 19], they are reported to be absent in some breastfed infants [20]. In such infants, bifidobacteria may have been absent during a critical colonization window [21], e.g. establishment of bifidobacteria is reported to be delayed or absent in infants born via caesarean section [10, 22]. Thus, bifidobacteria are used as probiotics to encourage the development of a healthy gut microbiota [23–25]. However, the efficacy of exogenously administered bifidobacteria depends on its relationship with the resident gut microbiota [26], and persistent colonization remains an issue [27, 28]. Therefore, elucidating mechanisms influencing the formation of bifidobacterial communities, in particular the role of assembly history and priority effects, may guide future strategies for microbiota-based therapeutic interventions, as well as explain differential establishment of bifidobacterial communities within the infant gut.

Here, we investigated the influence of assembly history on the compositional structure of infant gut-associated bifidobacterial communities. The four bifidobacterial species used in this study, *Bifidobacterium bifidum* JCM 1254*, Bifidobacterium breve* UCC2003*, Bifidobacterium longum* subspecies *longum* MCC10007 (*B. longum*), and *Bifidobacterium longum* subspecies *infantis* ATCC 15697 ^T^ (*B. infantis*), employ species-specific HMO assimilation strategies, which were predicted to dictate competitive outcomes. In vitro cultivation experiments in HMO-containing medium showed that assembly history significantly influenced community structure. Our results revealed that despite its limited HMO-assimilation ability, *B. breve* dominated through priority effects, which were partly underpinned by its ability to utilize fucose, an HMO degradant produced by other species. We also analysed publicly available in vivo faecal metagenome data from a cohort of infants and observed trends consistent with our in vitro findings, particularly for *B. breve*. Together, our study demonstrates the importance of priority effects on the establishment of bifidobacterial communities.

## Results

### Genome-based predictions and monoculture characterization of infant gut-associated bifidobacterial strains

**Fig. 1:**
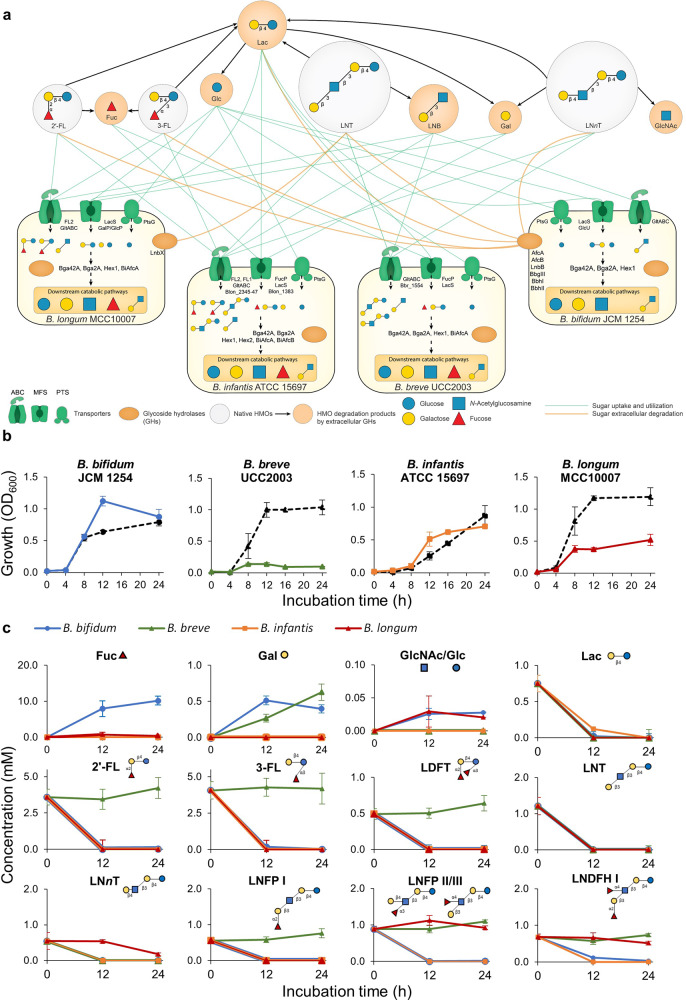
Genomic and monoculture characterization of infant gut-associated bifidobacteria used in this study. **a** Schematic summary of predicted HMO-utilization phenotypes of each bifidobacterial strain. The HMO-utilization phenotypes of each bifidobacterial strain (*B. bifidum* JCM 1254, *B. breve* UCC2003, *B. infantis* ATCC 15697 ^T^, and *B. longum* MCC10007) were characterized based on genomic predictions. Each strain is shown with its respective transporters and GHs (see Supplementary Table 2). The utilization pathways for four representative HMO molecules (2′-FL, 3-FL, LNT, and LN*n*T) and their mono- and di-saccharide degradants (Lac, LNB, Fuc, Glc, Gal, GlcNAc) are shown. The green lines indicate sugar uptake and utilization by each strain. The orange lines connect extracellular bacterial enzymes with the substrates. The black arrows connect HMO molecules with their degradants after extracellular degradation. **b** Monoculture growth curves of each strain grown in HMO-supplemented YCFA medium. Growth of each strain was monitored by measuring OD_600_ at each time point. All strains were also grown in Lac as a positive control, shown as black dotted lines. Data showing the total OD_600_ represent averages and error bars represent ± standard deviation of biological quadruplicates. **c** HMO-utilization profiles of each strain. Culture supernatant was collected at each time point. The remaining sugars in the medium were labeled with 2-AA and analyzed by HPLC (as described in the Materials and Methods section). The consumption of each sugar by each strain is indicated by different colors (blue: *B. bifidum*, green: *B. breve*, orange: *B. infantis*, red: *B. longum*). Data represent averages and error bars represent ± standard deviation of biological quadruplicates. It should be noted that LNB was not detected at the indicated time points.

Four infant gut-associated *Bifidobacterium* strains were used in this study: *B. bifidum* JCM 1254, *B. breve* UCC2003*, B. infantis* ATCC 15697 ^T^, and *B. longum* MCC10007. The genome of *B. bifidum* JCM 1254 was *de novo* sequenced in this study. We combined genomics-based (in silico) metabolic reconstruction with in vitro culturing to assess the HMO utilization capabilities of these strains. Our HMO mixture, purified from pooled breastmilk, included the following oligosaccharides: 2′-fucosyllactose (2′-FL), 3-fucosyllactose (3-FL), lactodifucotetraose (LDFT), lacto-*N-*tetraose (LNT), lacto-*N-neo*tetraose (LN*n*T), lacto-*N-*fucopentaose (LNFP) I, LNFP II/III, and lacto-*N-*difucohexaose (LNDFH) I (Supplementary Table 1). Using a subsystem-based approach implemented in the SEED genomic platform [29] we first established the presence/absence of genes encoding transporters and catabolic pathways for HMO constituents/degradation products (oligosaccharides: lacto-*N*-triose II (LNTri II), lacto-*N*-biose (LNB), and lactose (Lac); and their monosaccharide components: glucose (Glc), galactose (Gal), *N*-acetylglucosamine (GlcNAc), fucose (Fuc)) (see Supplementary Table 2). This analysis included repertoires of corresponding glycoside hydrolases (GHs) and transporters involved in HMO utilization in each of the four *Bifidobacterium* strains (Fig. 1a).

Genomic analysis revealed that the strains used in this study encode varying sets of GHs and transporters for HMO utilization, reflecting different strategies implemented by these *Bifidobacterium* strains. Overall, *B. infantis* ATCC 15697 ^T^ had the most expansive set of HMO transporters and corresponding intracellular GHs, whereas *B. bifidum* JCM 1254 had the highest number of extracellular GHs for HMO degradation [30]. While most of the purified HMOs used in our study are fucosylated, there was an apparent absence of the Fuc catabolic pathway in *B. bifidum* JCM 1254, which is a common trait of this species. *B. longum* MCC10007, *B. infantis* ATCC 15697 ^T^, and *B. breve* UCC2003 possess cytoplasmic Fuc catabolic enzymes, but only *B. infantis* ATCC 15697 ^T^ and *B. breve* UCC2003 encode the Fuc transporter FucP [31, 32] (Supplementary Table 2). All four strains were predicted to utilize lactose based on the presence of Lac permease LacS [33] and β-1,4-galactosidase Bga2A (GH2) [34] orthologs in all four genomes (Table 1, Supplementary Table 2).

**Table 1:**
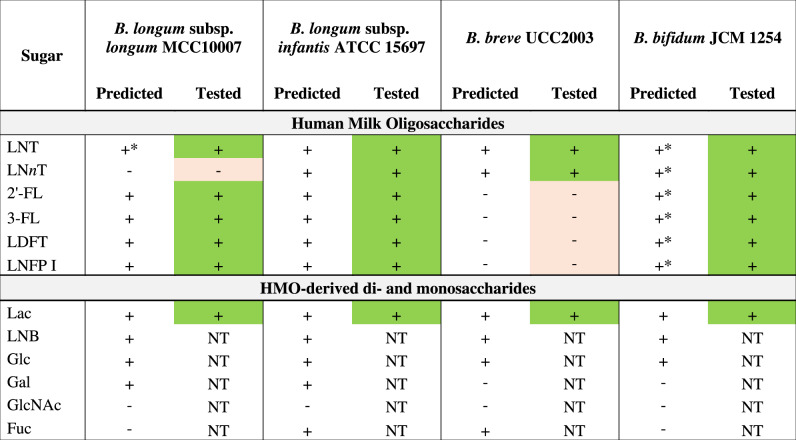
Predicted HMO/HMO degradation product utilization phenotypes of strains in the study.

+ = predicted utilizer (transporter, GH/catabolic enzyme genes are present) +* = predicted degrader (extracellular GH genes are present) - = predicted non-utilizer.

Colours: green = consumes a sugar; light red = does not consume a sugar; NT = not tested.

*LNT* Lacto-*N*-tetraose, *LNnT* Lacto-*N*-*neo*tetraose, *2*′*-FL* 2′-Fucosyllactose, *3-FL* 3-Fucosyllactose, *LDFT* Lactodifucotetraose, *LNFP* Lacto-*N-*fucopentaose, *Lac* Lactose, *LNB* Lacto-*N-*biose I, *Glc* Glucose, *Gal* Galactose, *GlcNAc* *N*-Acetylglucosamine, *Fuc* Fucose.

The HMO-consumption behaviour of these strains was evaluated in vitro through monoculture studies in a medium containing 1% (*w/v*) of either Lac (as the positive control) or a mixture of HMOs purified from pooled breastmilk as the sole carbon source (Fig. 1b), and sugar consumption was monitored using HPLC (Fig. 1c). Consistent with expectations, all strains grew well on Lac. With HMOs, strains predicted to encode the most expansive set of HMO-active GHs and associated HMO transporters, i.e., *B. bifidum* and *B. infantis*, showed substantial growth (final OD_600_ > 0.7). *B. longum* showed moderate growth (final OD_600_ > 0.5), while *B. breve* showed limited growth (final OD_600_ < 0.3) (Fig. 1b). Overall, carbohydrate consumption profiles were consistent with the phenotypes predicted from genomic data (Table 1): *B. bifidum* and *B. infantis* consumed all HMOs, *B. longum* consumed LNT and fucosylated HMOs (2′-FL, 3-FL, LDFT, and LNFP I), the latter of which is enabled by the presence of the fucosyllactose (FL) transporter (found in approximately 3 % of *B. longum* strains [16, 26]), and *B. breve* only utilized LNT and LN*n*T (Fig. 1c).

### Pairwise cultivation of bifidobacterial strains in HMO-supplemented medium

**Fig. 2:**
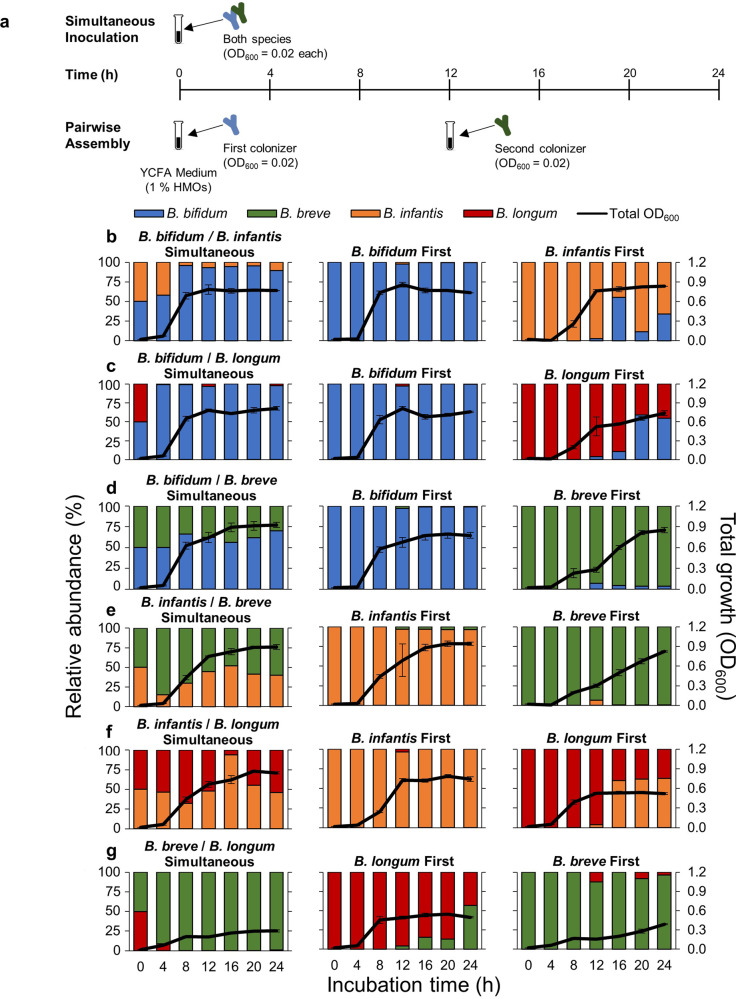
Pairwise cultivation of infant gut-associated bifidobacteria. **a** Experimental design. Pairwise culturing experiments were performed for all possible combinations. In simultaneous cultures, both species were introduced together into medium supplemented with HMOs at the beginning of the experiment (at 0 h). To test for the effect of assembly history, the first species was inoculated at the beginning of the experiment, and the second species was inoculated 12 h later. **b**–**g** Culturing results for each pair. Total OD_600_ indicated by the black line, was measured at each time point, and the relative abundance of each species was quantified using qPCR. The relative abundance of each strain is indicated by different colors (blue: *B. bifidum*, green: *B. breve*, orange: *B. infantis*, red: *B. longum*). Relative abundances at 0 h are calculated theoretical values. Data showing the total OD_600_ represent averages and error bars represent ±standard deviation of biological quadruplicates. The bar graphs showing the relative abundances of each species show averages of biological quadruplicates. **b** *B. bifidum* and *B. infantis* pair. **c** *B. bifidum* and *B. longum* pair. **d** *B. bifidum* and *B. breve* pair. **e** *B. infantis* and *B. breve* pair. **f** *B. infantis* and *B. longum* pair. **g** *B. breve* and *B. longum* pair.

To determine if priority effects influence the outcome of competition among bifidobacterial species, we performed all possible pairwise cultivations (Table 2). Here, we consider any significant effect of arrival order on the absolute and relative abundances of each species as priority effects. Priority effects can be either facilitative, in which the early arriving species positively affects the later arriving species (e.g., changing the types of resources available and enabling later arriving species to colonize), or inhibitory, in which the early arriving species negatively affects the later arriving species (e.g., using up resources and making them unavailable for later arriving species) [6]. For each pair, one species was inoculated into the culture medium first, and the second species was inoculated 12 h later. As a control, both species were also inoculated into the medium simultaneously (Fig. 2a). Experimental duration was 24 h so that all species had at least 12 h in the medium after inoculation (at which growth plateaus during monoculture (Fig. 1b)), and sugars in the medium were depleted in most assembly sequences (Extended Data Figs. 1–4). Pairwise growth curves are summarized in Fig. 2b–g, and a species was considered dominant if its relative abundance was >50 % of the community. Furthermore, we quantified the strength of priority effects using the equation proposed by Vannette and Fukami [35], by comparing a given species’ absolute abundance when it was the first colonizer with its absolute abundance when it was the second, invading species (Supplementary Table 3).

**Table 2:**
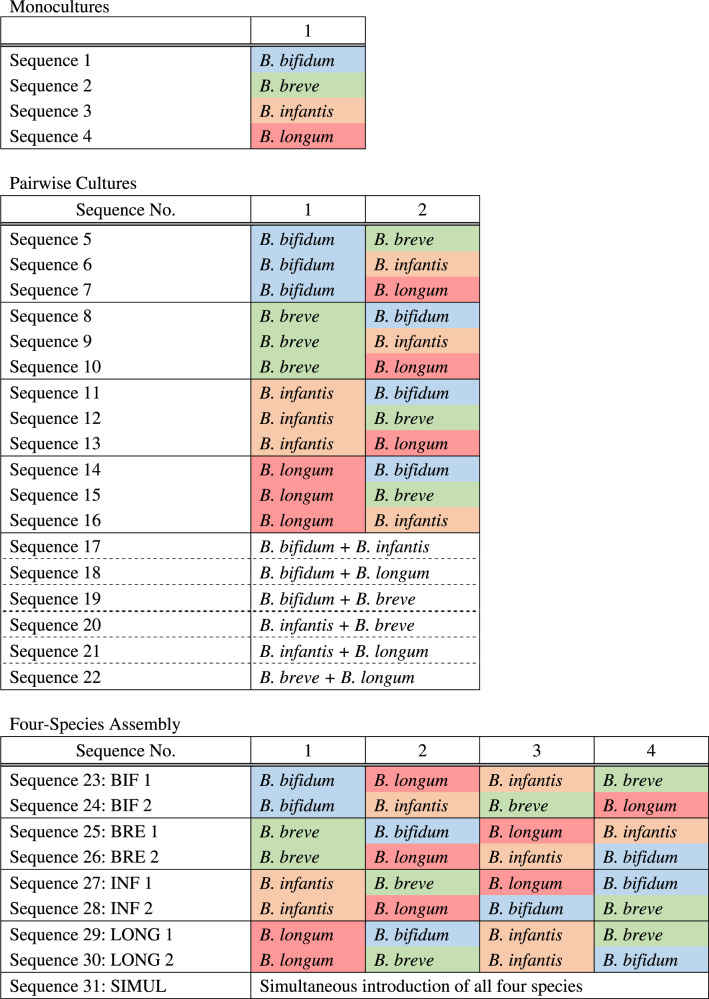
Community assembly sequences used in this experiment.

When *B. bifidum* JCM 1254 was cultivated together with *B. infantis* ATCC 15697 ^T^, the early arriving species dominated through inhibitory priority effects (Fig. 2b). As *B. bifidum* and *B. infantis* are both capable of utilizing all types of HMOs, they both depleted most of the available resources within 12 h when inoculated first (Extended Data Figs. 1, 2). Priority effects were stronger for *B. bifidum* (Supplementary Table 3), and it was a stronger competitor overall as it dominated in simultaneous culture and also maintained a stable population even when *B. infantis* was first (Fig. 2b). For the *B. bifidum* and *B. longum* pair, the final relative abundance for *B. bifidum* was greater regardless of arrival order (Fig. 2c). However, priority effects and significant growth were observed for *B. longum* when it was the first colonizer, possibly because it was able to utilize the HMO-degradants produced by *B. bifidum* (Fig. 2c, Supplementary Table 3).

For the *B. breve* and *B. bifidum* pair*, B. bifidum* dominated in simultaneous culture and when it was inoculated first (Fig. 2d). When *B. breve* was first, priority effects and substantial growth were observed, as it dominated and drove *B. bifidum* to near extinction (Fig. 2d, Supplementary Table 3) despite its limited HMO-consumption ability and low growth in HMO-supplemented medium during monoculture (Fig. 1b). However, *B. breve* growth remained low until *B. bifidum* inoculation, upon which *B. breve* showed biphasic growth (Fig. 2d). A similar pattern was observed for the *B. breve* and *B. infantis* pair, in which priority effects were observed for *B. breve* (Supplementary Table 3) and it outcompeted *B. infantis* if inoculated first. However, *B. breve* growth remained low until *B. infantis* arrival (Fig. 2e), suggesting that the presence of *B. bifidum* and *B. infantis* had a facilitative effect on *B. breve* growth.

In the *B. infantis* and *B. longum* pair, *B. infantis* generally dominated regardless of arrival order (Fig. 2f). However, priority effects were observed for *B. longum* (Supplementary Table 3) and it was able to maintain a stable population even after *B. infantis* inoculation (Fig. 2f). As for the *B. breve* and *B. longum* pair, the average final total OD_600_ was low in all assembly sequences (Simultaneous: 0.30, *B. longum* first: 0.50, *B. breve* first: 0.37) and priority effects were observed for neither species (Supplementary Table 3), but *B. breve* dominated in all scenarios by 24 h (Fig. 2g). Furthermore, while HMOs were completely consumed in most pair combinations by the end of the cultivation period, oligosaccharides with longer chains (LNFP II/III and LNDFH I) remained in the spent medium of *B. breve* and *B. longum* cultures (Extended Data Figs. 1–4), which was consistent with their predicted HMO assimilation phenotypes.

### Four-species assemblages in HMO-supplemented medium

**Fig. 3:**
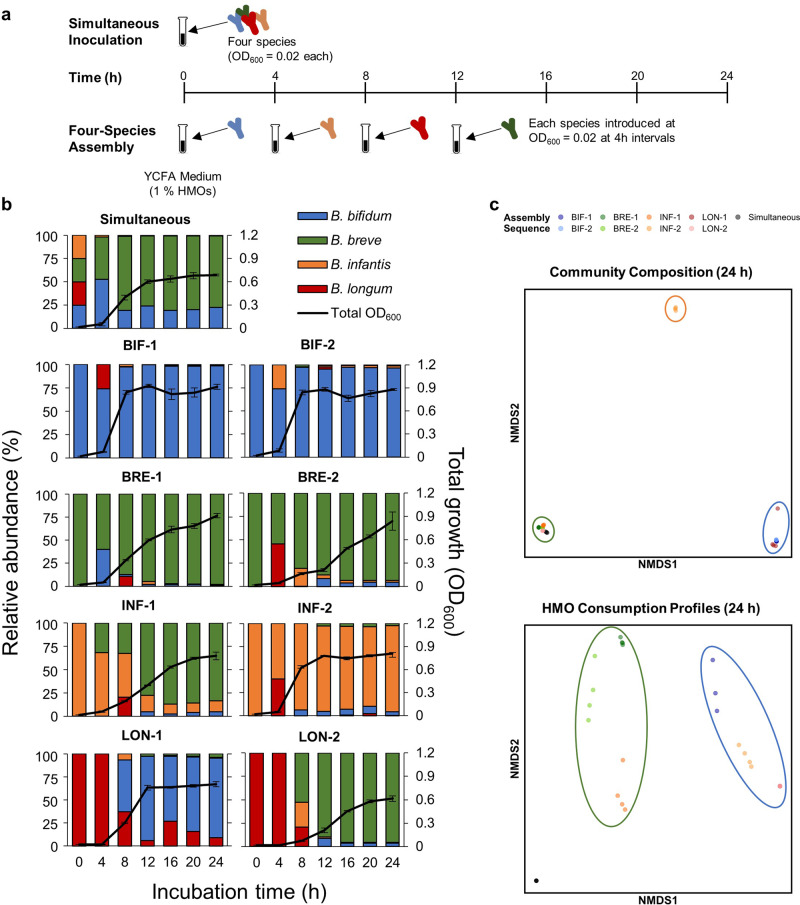
Results of four-species assemblages. **a** Experimental design. Culturing experiments were performed with all four species, and assembly order was manipulated. Assembly history sequences are summarized in Table 2. As a control, all four species were introduced into medium supplemented with HMOs at the beginning of the experiment (Simultaneous). **b** Culturing results. Total OD_600_, indicated by the black line, was measured at each time point, and the relative abundance of each species was quantified using qPCR. Data represent averages of biological quadruplicates, and error bars indicate ± standard deviation. The relative abundance of each strain is indicated by different colors (blue: *B. bifidum*, green: *B. breve*, orange: *B. infantis*, red: *B. longum*). **c** NMDS plots based on the community composition and HMO consumption profiles at 24 h. Each color represents a different assembly sequence. In the top panel, three clusters form based on the final community composition: blue (*B. bifidum* dominant communities), green (*B. breve* dominant communities), and orange (*B. infantis* dominant communities). In the bottom panel, two clusters based on the HMO consumption profiles form: blue (communities with high amounts of fucose remaining) and green (communities with 3-FL and LNDFH I unconsumed) circles.

Assembly history was manipulated with all four bifidobacterial strains. As a control, all four species were inoculated simultaneously at the beginning of the experiment (Fig. 3a). Eight assembly sequences were selected so that each species was inoculated first, twice. The second, third, and fourth species were randomly selected (Table 2). Data were collected for 24 h so that all species were in the medium for at least 12 h after inoculation. At 24 h, most HMOs in the medium were depleted, except 3-FL and LNDFH I in the INF-1, BRE-1, and BRE-2 sequences (Extended Data Fig. 5).

*B. breve* (average relative abundance: 77.5%) was the dominant species in simultaneous culture, followed by *B. bifidum* (average relative abundance: 21.9%) and *B. infantis* (average relative abundance: 0.6%) at 24 h, while *B. longum* was below the detection limit and presumed to be extinct within 4 h (Fig. 3b). For communities in which *B. bifidum* was introduced first (BIF-1 and BIF-2 sequences), *B. bifidum* was the dominant species with a final OD_600_ of 0.90 and 0.85, respectively. *B. breve* also dominated when it was inoculated first, and the community profile and final OD_600_ were comparable between the two sequences (BRE-1 and BRE-2). However, the growth curves of the BRE-1 and BRE-2 sequences (OD_600_) were dependent on the identity of the second species. While the average total OD_600_ reached 0.74 at 12 h when *B. bifidum* was second (BRE-1), it was 0.28 when *B. longum* was second (BRE-2). In assembly sequences in which *B. infantis* was first, *B. infantis* dominated when *B. longum* was second (INF-2). However, *B. infantis* was outcompeted by *B. breve* in the final community at 24 h when *B. breve* was second (INF-1). With regards to *B. longum*, not only was it unable to become the dominant competitor in all assembly sequences, but it was also undetectable by 24 h in all sequences except for LON-1, where *B. longum* was inoculated first and followed by *B. bifidum*.

Nonmetric multidimensional scaling (NMDS) based on the final community structure at 24 h revealed three different possible community outcomes: 1) *B. breve* dominant, 2) *B. bifidum* dominant, and 3) *B. infantis* dominant (Fig. 3c). Further analysis with permutational multivariate analysis of variance (PERMANOVA) based on Bray-Curtis distances revealed that assembly history had a significant influence on final community structure (F_8,27_ = 64.28, *p* < 0.001), and the identity of the first species (Partial *R*^2^ = 0.91, *p* < 0.001) and the second species (Partial *R*^2^ = 0.04, *p* < 0.001) determined the final community outcome.

Regression analysis was performed to quantify the strength of priority effects. Calculations were performed to predict final population abundance of each species based on arrival order. A significant negative relationship was found for *B. breve* (F_1,30_ = 335.3, *p* < 0.001, β = −0.29, *R*^2^ = 0.92), *B. bifidum* (F_1,30_ = 30.94, *p* < 0.001, β = −0.24, *R*^2^ = 0.51), and *B. infantis* (F_1,30_ = 6.615, *p* = 0.015, β = −0.12, *R*^2^ = 0.18), indicating that the earlier these three species were introduced into the community, the higher its final abundance. However, no significant relationship was found for *B. longum* (F_1,30_ = 1.811, *p* = 0.177, β = −0.01, *R*^2^ = 0.03).

We also examined the HMO-consumption profiles of each assembly sequence (Extended Data Fig. 5). NMDS and PERMANOVA based on the final HMO-consumption profiles at 24 h showed that the amount of LNDFH I, Fuc, 3-FL, and LNFP II/III remaining in the medium significantly contributed to the variation among communities (Fig. 3c, Supplementary Table 4). Two main clusters formed, one of which contained communities dominated by *B. bifidum* with high amounts of fucose remaining in the spent medium, while the second contained communities dominated by *B. breve*, in which 3-FL and LNDFH I remained unconsumed (Extended Data Fig. 5).

### Metagenomic data mining analysis

**Fig. 4:**
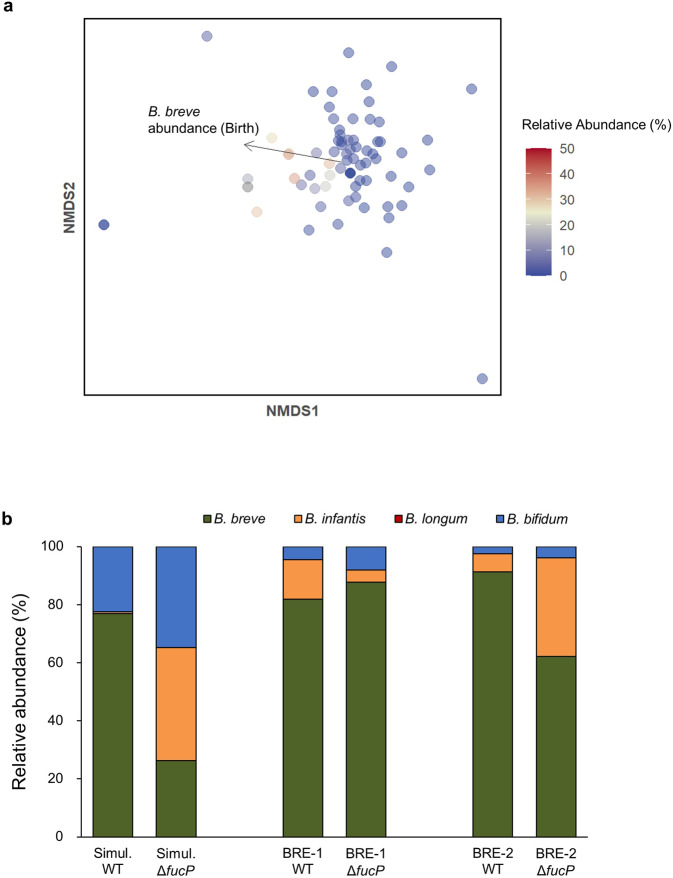
In silico data mining and in vitro culturing analyses demonstrate that *B. breve* benefits from priority effects. **a** Analysis of in vivo microbiome data. Taxonomic identification was performed using Kraken2 v2, and species abundances were estimated using Bracken v2.6.1. Nonmetric multidimensional scaling (NMDS) ordination plots of bifidobacterial community data from the guts of 4-month old infants who were at least partially breastfed (exclusively breastfed or mixed fed) at birth and at 4 months of age (*n* = 73) (see Extended Data Fig. 6 and Supplementary Table 5). Each point corresponds to one individual. The color gradient indicates the *B. breve* abundance within the total gut microbiota at 4 months of age. Statistically significant loadings are indicated as black arrows. **b** Culturing results using the Δ*fucP* mutant of *B. breve* UCC2003. Sequences of 4-species culturing in which *B. breve* dominated was used, and the experiment was repeated with the wild type (WT) and Δ*fucP* mutant of *B. breve* UCC2003. Relative abundance of each species was quantified using qPCR, and each strain is indicated by different colors (blue: *B. bifidum*, green: *B. breve*, orange: *B. infantis*, red: *B. longum*). Data represent averages of biological triplicates.

We further analysed bifidobacterial communities in a cohort of European infant-mother pairs from a study performed by Bäckhed et al. [36]. This metagenomic dataset followed the infant gut microbiota at 0, 4, and 12 months of age, as well as the mother’s gut microbiota at the time of delivery. The microbiotas of 73 infants who were at least partially breastfed (exclusively breastfed or mixed fed) at both 0 and 4 months of age were analysed. By 12 months of age, the majority of the infants had been weaned and the average relative abundance of bifidobacteria was 2.46% (Extended Data Fig. 6). PERMANOVA revealed that the relative abundance of *B. breve* in the infant gut microbiota at birth contributed to the bifidobacterial community structure at 4 months (Partial *R*^2^ = 0.09, *p* = 0.0499; Fig. 4a, Supplementary Table 5). However, such significance was not observed for *B. bifidum*, the *B. longum* group, and other species. We also assessed whether the presence of a species at birth is associated with its dominance (>50% of the bifidobacterial community) at 4 months using Fisher’s exact test. Based on this analysis, we found that the presence of *B. breve* at birth (40 out of 73 infants) was associated with its dominance at 4 months (*p* = 0.029), while such patterns were not observed for *B. bifidum* or the *B. longum* group. However, correlation analyses showed that at birth and 4 months, there was a significant correlation between the abundance of *B. bifidum* and the total abundance of *Bifidobacterium* species (Birth: Pearson’s *r* = 0.33, *p* = 0.004; 4 months: Pearson’s *r* = 0.43, *p* = 0.0002). Additionally, the presence of *B. bifidum* was positively correlated with *B. longum* (Pearson’s *r* = 0.27, *p* = 0.02) at birth. The stimulatory effect of *B. bifidum* on the growth of other bifidobacterial species was observed in our in vitro pairwise and four-species assembly cultivation experiments (Figs. 2b, 3c), which is consistent with previous work that reported that the presence of *B. bifidum* in breastfed infant guts is associated with a higher prevalence of bifidobacteria at genus level [10].

### Fucose utilization ability of B. breve contributes to its dominance in vitro

*B. breve* dominated if present in the community early (Figs. 2d, e, g, 3b), despite its limited growth in HMO medium during monoculture (Fig. 1b, c) and the prevalence of genes for utilizing fucosylated HMOs is low (<13 %) among sequenced *B. breve* strains [26] (Fig. 1b, c). In our in vitro system, we found that Fuc was consumed in *B. breve* dominant communities, while left unconsumed when other species dominated (Fig. 3c, Extended Data Fig. 5). Furthermore, *B. breve* UCC2003 and all *B. breve* strains (46 out of 46 strains whose complete genomes are reported in the NCBI database as of October 27, 2021) possess homologs of the Fuc transporter FucP (Bbr_1742) [31] (Supplementary Table 2). Therefore, we compared wild-type (WT) and Δ*fucP* strains of *B. breve* UCC2003 to examine whether its ability to import fucose contributed to its persistence and/or dominance. We selected sequences in which *B. breve* dominated (Simultaneous, BRE-1, BRE-2; Table 2) and repeated the culturing experiments. Compared to when the WT strain was used, the average relative abundance of *B. breve* decreased from 76.9% to 26.3% (Δ50.6 %; Student’s two-tailed *t*-test, *p* = 0.024) in the simultaneous sequence and from 91.3% to 62.2% (Δ29.1 %; Student’s two-tailed *t*-test, *p* = 0.027) in the BRE-2 sequence (Fig. 4b). No significant change was observed in the BRE-1 sequence (Student’s two-tailed *t*-test, *p* = 0.08), in which *B. bifidum* is inoculated after *B. breve*. Furthermore, analysis of culture supernatant showed that while fucose accumulated in sequences using *B. breve* Δ*fucP*, it was depleted in sequences with WT *B. breve* (Extended Data Fig. 7). These results strongly suggest that the ability to utilize fucose contributed to the competitive ability of *B. breve* within bifidobacterial communities. This contrasts with *B. infantis*, which leaves Fuc unconsumed when co-cultured with other species, even though it also possesses FucP (Extended Data Fig. 5b, INF-2).

## Discussion

Using infant gut-associated bifidobacterial communities in HMO-supplemented environments, we experimentally controlled the order of species arrival to evaluate the effect of assembly history and priority effects. Four strains that display a variety of species-specific mechanisms for HMO assimilation were used. Based on the predicted phenotypes and monoculture data, we hypothesized that in HMO environments, (1) *B. bifidum* JCM 1254 and *B. infantis* ATCC 15697 ^T^ would be strong competitors due to their ability to assimilate a variety of HMOs through extracellular and intracellular GHs, respectively, (2) *B. longum* MCC10007 would be a moderate competitor, as it cannot consume LN*n*T, but can consume LNT and specific fucosylated sugars such as 2′-FL, 3-FL, LDFT, and LNFP I, and (3) *B. breve* UCC2003 would be a weak competitor as its HMO-consumption ability is limited to LNT and LN*n*T (Fig. 1c; Supplementary Table 2). Here, we found that strong competitors like *B. bifidum* and *B. infantis* can dominate through inhibitory priority effects in vitro, in which the early arriving species apparently depletes resources for later arriving species. On the other hand, *B. breve*, despite its limited HMO-utilization ability, benefitted from facilitative effects by utilizing fucose and other degradants provided by competitors like *B. bifidum* and *B infantis*. Our results highlight the importance of early arriving species during community assembly, whose mechanisms are influenced by species-specific competitive strategies and sugar consumption phenotypes.

Regarding *B. bifidum* and *B. infantis*, inhibitory priority effects were observed in both pairwise and four-species assemblages (except the INF-1 sequence), and communities behaved as would be predicted from genomic data and assembly order. For example, in the *B. bifidum* and *B. infantis* pairwise culture, the early-arriving species became dominant (Fig. 2b), and the outcome of competition was dependent on which species arrived and acquired the resources first [6]. The results of the four-species assemblages also showed that assembly history had a significant effect on the final community structure. A negative relationship between arrival order and final abundance is indicative of inhibitory priority effects [37] and regression analyses of 4-species assemblages showed that such negative relationships were observed for *B. bifidum* and *B. infantis*. In *B. bifidum-* and *B. infantis-*dominant communities, most of the carbohydrates in the medium were depleted soon after inoculation (Extended Data Figs. 1, 2, 5), suggesting that *B. bifidum* and *B. infantis* dominated through niche pre-emption. Furthermore, different assembly sequences gave rise to three different community states (Fig. 3c), a predicted consequence of priority effects [6, 38, 39]. PERMANOVA revealed that the identity of the first and second colonizers primarily determined the community outcome, highlighting the significant effect early arriving species have on the final community. For instance, in communities in which *B. infantis* was inoculated first (INF-1, INF-2), *B. infantis* dominated when followed by *B. longum* (INF-2; Fig. 3b). However, it was unexpectedly outcompeted when *B. breve* was inoculated second (INF-1; Fig. 3b).

**Fig. 5:**
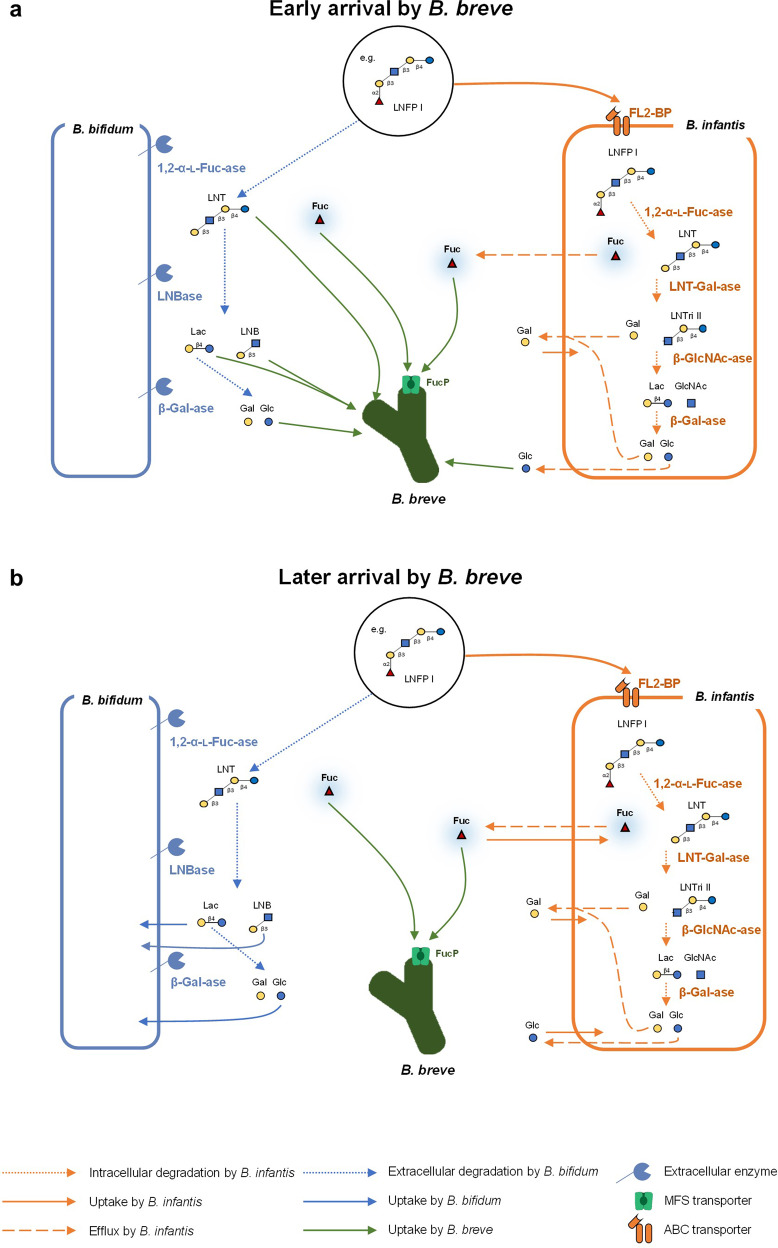
Predicted HMO-utilization pathways that enable *B. breve* to benefit from priority effects. The HMO-utilization pathways, with LNFP I as a representative example, are shown for *B. bifidum, B. infantis*, and *B. breve*. Similar mechanisms are expected for other fucosylated HMOs. Activity, enzymes, and transporters attributable to *B. bifidum* are shown in blue, *B. infantis* in orange, and *B. breve* in green. Solid lines indicate assimilation by each species, round-dot lines indicate enzymatic degradation, and dashed lines indicate efflux. *B. breve* alone cannot assimilate LNFP I. **a** When *B. breve* is the prior colonizer, it is ready to utilize degradants as soon as they are made available by either *B. bifidum* or *B. infantis*. **b** When *B. breve* is the latter colonizer, most of the degradants are consumed before *B. breve* is introduced. LNT Lacto-*N*-tetraose; LNFP, Lacto-*N-*fucopentaose; LNTri II, Lacto-*N-*triose II, Fuc, Fucose; Glc, Glucose; GlcNAc, *N*-Acetylglucosamine; Gal, Galactose.

The behaviour of *B. breve* was not in accordance with our initial expectations and the facilitative effects of other taxa were observed in our in vitro experiments. Despite considerable fitness differences predicted based on HMO-utilization ability, *B. breve* was able to dominate against stronger competitors (*B. bifidum* and *B. infantis*) in both pairwise and 4-species assemblages if inoculated early (Figs. 2d, e, g, 3b). While most of the carbohydrates in the medium in *B. bifidum-* and *B. infantis-*dominant communities were depleted, (Extended Data Figs. 1, 2, 5), sugars such as 3-FL and LNDFH I were left unconsumed in *B. breve-*dominant communities (Extended Data Fig. 5c). The later-arriving strains that can utilize those sugars were unable to outcompete *B. breve*, suggesting *B. breve* does not dominate through niche pre-emption. One possible explanation is the differential fucose-utilization phenotypes among infant gut-associated bifidobacteria, which allows *B. breve* to proliferate through priority effects (Fig. 5). In the INF-1 sequence, in which *B. breve* is introduced second after *B. infantis*, fucose is present in the medium at 12 h and decreases as *B. breve* abundance increases (Fig. 3b, Extended Data Fig. 5b). During pairwise culture, *B. breve* growth was significantly enhanced when co-cultured with *B. bifidum* or *B. infantis* (Fig. 2d, e), both of which liberate fucose and other monosaccharides during HMO metabolization [40]. With *B. bifidum*, fucose is released through extracellular degradation and left unconsumed [30, 41, 42]. Therefore, niche differentiation may be helping *B. breve* to maintain a competitive presence when cultured with *B. bifidum*. On the other hand, *B. infantis* temporarily excretes fucose and other monosaccharides after intracellular degradation of HMOs, possibly to counteract increasing internal osmotic pressure, and re-imports them later [40]. In co-culture with *B. infantis*, the timing of arrival becomes crucial — for *B. breve* to utilize fucose, it must be present in the community after *B. infantis* temporarily excretes fucose, but before *B. infantis* re-imports fucose (Fig. 5). Indeed, when the cultivation experiments were performed with *ΔfucP B. breve*, an isogenic derivative of *B. breve* UCC2003, the relative abundance of *B. breve* decreased significantly (Fig. 4b), supporting the fact that fucose (metabolism) enhances the competitive ability of *B. breve*.

Bifidobacterial communities in the gut of a breastfed infant often fall into one of two groups: *B. longum-*dominant or *B. breve-*dominant [10, 43–45]. A variety of factors such as delivery mode, feeding regime, and maternal microbiota can affect the assembly of bifidobacteria in the infant gut, and the ecological mechanisms that lead to *B. longum* dominance are yet to be determined. With regards to *B. breve*, only a limited subset of strains can utilize fucosylated HMOs [26], and in general, its HMO-consumption ability is limited to LNT and LN*n*T. Therefore, factors that contribute to *B. breve* dominance have not been well understood. Here, an analysis of an in vivo metagenomic dataset revealed that if *B. breve* was present in the infant gut microbiota at birth, it was more likely to dominate in the community at 4 months of age (Fig. 4a, Extended Data Fig. 6), and our in vitro results suggest that fucose can provide *B. breve* a competitive advantage. We do note that *B. breve*, as well as *B. infantis* (another species that possesses FucP), does not grow well on fucose alone [46]. A study using *B. infantis* ATCC15697^T^ found that compared to growth on individual sugars, growth significantly increased when the strain was presented with excess fucose in combination with a preferred substrate [47]. Therefore, while fucose alone is not an efficient energy source, it may be serving as a molecule that enhances competitiveness when combined with other sugars. However, continued research is needed to understand the mechanisms by which fucose promotes bifidobacterial growth.

The role of priority effects in the development of the infant gut microbiota is gaining interest, and breastfeeding is a key factor that can significantly influence ecological interactions [48]. Therefore, we focused on bifidobacteria, which are among the first colonizers of the infant gut and utilizers of HMOs found in breastmilk. Here, we show that their sugar-utilization phenotypes determined the outcomes of competition and we have also identified several future research needs. For example, the physiological effects different bifidobacterial species have on infant health has yet to be elucidated. Infant-type bifidobacteria produce indole lactic acid (ILA), an anti-inflammatory molecule that helps regulate intestinal homeostasis in the infant host, and the ability of ILA production is linked with HMO assimilation ability [49, 50]. While the ILA production ability is widespread among infant-type species, only fucose-utilizing species like *B. breve* also produce 1,2-propanediol, which is utilized by other gut commensals such as *Eubacterium hallii* to produce propionate [51–55]. Additionally, the presence of *B. bifidum* is associated with a higher population abundance of *Bifidobacterium* at the genus level [10]. Therefore, the identity of the dominant bifidobacterial species may affect host health both directly and indirectly through interactions with other taxa, and future work should address the effect of other gut commensals. While *Bifidobacterium* is often dominant in breastfed infant guts [10], several *Bacteroides* species are known to utilize HMOs [56]. *Bacteroides* have been reported to dominate in the absence of bifidobacteria [57], and mutual exclusion may be occurring through the depletion of HMOs. Furthermore, a study comparing infants from different European countries found that while both Russian and Finnish infants were breastfed (199 days and 268 days on average, respectively), *Bacteroides* remained the dominant taxa in Finnish infants and *Bifidobacterium* in Russian infants [58], suggesting that once established, community structure is not easily affected by continued dispersal from external sources such as different maternal body sites, home environment, and breastmilk [8, 59–62]. Additionally, a study that administered probiotic *B. breve* to low birth weight infants suggests that the infant gut microbiota is established within 24 h of birth [63]. In that study, a bifidobacteria-rich microbiota formed earlier when the first dose of *B. breve* was administered a few hours after birth, as compared to when administered more than 24 h after birth, indicating that the first 24 h of life is a crucial colonization window for gut microbes. To further determine the extent to which priority effects can inform probiotic therapies, additional work that closely mimics the infant gut environment are needed.

As in many other experimental studies, we cannot exclude the possibility that our results are specific to the strain and medium we used, as HMO utilization phenotypes of bifidobacterial species can vary within a species [26]. Additional work utilizing multiple strains from each species, as well as strains isolated from infant faeces could further elucidate the role of priority effects within bifidobacterial communities. However, with regards to *B. breve*, all known strains possess homologs of FucP and the fucose catabolic pathway, suggesting the possibility that fucose may enhance the competitive ability of other *B. breve* strains as well. Therefore, our results provide insight into previously unexplained *B. breve* abundance in breastfed infants [10, 43–45]. Despite certain limitations, we demonstrate the prevalence of priority effects in bifidobacterial communities, particularly for *B. breve*, and found that early introduction by just a few taxa has the potential to divert the maturational trajectory of the microbial community. Our results suggest that long-term persistence of bifidobacteria and other probiotic strains might be achieved if interventions in the infant gut microbiota are implemented during the early stages of community assembly. As several HMO species are becoming more commercially available in large quantities, additional work using chemostat studies and animal models could provide further insight into how the timing and use of probiotic species can help selectively manipulate the gut microbiota in a way that is both predictable and advantageous to provide disease protection and health-promoting effects for the infant host.

## Materials and methods

### Chemicals

Fuc, Glc, Gal, 2-AA (anthranilic acid), and sodium cyanoborohydride were purchased from Nacalai Tesque (Kyoto, Japan). GlcNAc and Lac were purchased from Wako Pure Chemical Industries (Osaka, Japan). LN*n*T and LNFP I were purchased from Dextra Laboratory (Reading, UK). 2′-FL, 3-FL, LDFT, and LNT were obtained from IsoSep (Tullinge, Sweden), or provided as gifts from Glycom A/S (Hørsholm, Denmark). LNFP II, LNFP III, and LNDFH I were purchased from Carbosynth (Berkshire, UK), and isomaltoheptaose was purchased from Seikagaku Kogyo (Tokyo, Japan). LNB was synthesized as described previously [64]. All reagents employed in this study were of analytical grade.

### Preparation of oligosaccharides from human milk

Milk samples were collected from healthy Japanese mothers who had not taken any antibiotics for at least 1 month before collection at Nagao Midwife Clinics (Kyoto, Japan). Informed consent was obtained from all mothers. The study was reviewed and approved by the Ethics Committee of Kyoto University (R0046-1) and the University of Shiga Prefecture (71-3) and was performed per the Declaration of Helsinki. HMOs were purified from the collected human milk samples as described previously [40]. During the purification process, sialylated oligosaccharides were eluted near the void fractions as polymeric compounds, and therefore absent from the HMO mixture used in this study. Some amount of Lac, which is a contaminant derived from the HMO purification process, was also detected. The composition of the HMO mixture used in this study was determined by analysis with HPLC and is summarized in Supplementary Table 1.

### Bacterial strains and culture conditions in HMO-supplemented medium

*Bifidobacterium bifidum* JCM 1254 and *Bifidobacterium longum* subspecies *infantis* JCM 1222 (ATCC 15697 ^T^) (*B. infantis*) were obtained from the Japan Collection of Microorganisms (JCM; RIKEN BioResource Center, Japan). *Bifidobacterium breve* NCIMB 8807 (UCC2003) was obtained from the National Collection of Industrial Food and Marine Bacteria Ltd. (NCIMB; Aberdeen, UK). *Bifidobacterium longum* subspecies *longum* MCC10007 (*B. longum*) was obtained from the Morinaga Culture Collection (MCC, Morinaga Milk Industries, Co. Ltd., Zama, Japan). The Δ*fucP* mutant derivative of *B. breve* UCC2003 was constructed in a previous study [31]. All strains used in this study were stored in glycerol stocks and kept frozen at −80 °C until use. All strains were routinely grown in and pre-cultured in Gifu Anaerobic Medium (GAM) broth (Nissui Pharmaceutical, Tokyo, Japan) under anoxic conditions using the AnaeroPack system (Mitsubishi Gas Chemical Co., Tokyo, Japan) at 37 °C. Culturing experiments were performed in YCFA medium, a medium with yeast, casitone, and fatty acids [65], which is known for supporting the growth of the majority of gut commensal species [66]. As a sole carbon source, the medium was supplemented with 1% HMOs or Lac as a positive control (*w/v*). All cultures were incubated under anoxic conditions at 37 °C and growth was monitored by measuring OD_600_ at each time point.

### Whole genome sequencing

Genomic sequencing for *B. bifidum* JCM 1254 was performed by first extracting genomic DNA from the strain sub-cultured in de Mann, Rogosa, Sharpe (MRS) medium. The libraries were prepared using a Nextera XT DNA Library Prep Kit (Illumina Inc.) per manufacturer instructions. Paired-end sequencing (29-fold coverage) was conducted on a MiSeq platform (Illumina Inc.) using MiSeq v3 Reagent Kit. Quality trimming of raw reads and de novo genomic assembly were performed in CLC Genomics Workbench (version 8.0) (Qiagen, Valencia, CA, USA) with default settings. Contigs with less than 100 nucleotides were removed. The open reading frame (ORF) predictions and annotations were performed with the DDBJ Fast Annotation and Submission Tool (DFAST) with the default settings [67]. Genomes for *B. breve* UCC2003 (Accession Number: NC_020517), *B. longum* MCC10007 (Accession Number: SAMN06368573), and *B. infantis* ATCC 15697 ^T^ (Accession Number: NC_011593) were retrieved from the NCBI GenBank public database (https://www.ncbi.nlm.nih.gov/genbank/).

### Bacterial genome annotation, in silico reconstructions, and phenotype predictions

In-depth genomic analysis of *Bifidobacterium* genomes was performed in the web-based mcSEED (microbial communities SEED) environment, a private clone of the publicly available SEED platform [68]. Genomes of *B. longum* subsp. *longum* MCC10007, *B. longum* subsp. *infantis* ATCC 15697 ^T^, *B. breve* UCC2003, and *B. bifidum* JCM 1254 were first annotated using RAST [68] and then imported into mcSEED. Subsystem-based, context-driven functional assignments of genes combined with manual curation were used to reconstruct and analyse the representation of HMO and HMO constituent utilization pathways in the selected genomes. Data on functional elements (transporters, glycoside hydrolases, downstream catabolic enzymes) involved in bifidobacterial HMO metabolism (Supplementary Table 2) was collected by the following: (1) extensive literature search using PaperBLAST;[69] (2) exporting information from the Carbohydrate Active enZyme (CAZy) [70] and Transporter Classification (TCDB) [71] databases.

### Experimental design

To test for priority effects in infant-gut associated bifidobacterial communities, culturing was performed in three different phases: monocultures, pairwise cultures, and four-species assemblages. For pairwise cultures and four-species assemblages, inoculation order was manipulated to examine its effect on community structure. For all culturing experiments, each strain was inoculated with an initial OD_600_ = 0.02 into 400 μL of YCFA medium containing 1 % HMOs as a sole carbon source, and sampling was performed every 4 h for 24 h. At each sampling time point, OD_600_ was measured to monitor growth. An aliquot of the culture medium was also collected and centrifuged. The supernatant was collected for sugar concentration analysis. The bacterial pellet was used for DNA extraction, which was performed using the phenol-chloroform method [72].

All cultivation experiments were performed in quadruplicate. Monoculture experiments for each strain were performed to characterize its growth in HMO-supplemented medium, as well as its sugar utilization profiles. Then, pairwise cultures were performed by allowing one species to colonize first and introducing the second species 12 h later, at which the growth curve of the first species plateaus. As a control, both species were inoculated simultaneously (Fig. 1a). All possible pairwise combinations were tested (Table 2; Pairwise). A series of four-species assemblages were also performed, in which 8 assembly sequences were selected so that each species was inoculated first, twice. The second, third, and fourth species were randomly selected. As a control, all four species were inoculated in the community simultaneously (Table 2; Four-Species Assembly). For four-species assemblages, each species was introduced at 4-hour intervals (Fig. 2a).

### Sugar concentration analysis

Culture supernatant was collected at each time point, clarified by centrifugation, and stored at −30 °C until use. For analysis with HPLC, the samples were thawed and mixed with isomaltoheptaose (internal standard). The sugars were fluorescence-labelled with 2-AA, and the reaction mixtures were desalted by solid-phase extraction as described previously [40, 73]. HPLC was performed using a Thermo U3000 HPLC system (Thermo Fisher Scientific, Waltham, MA). This was equipped with a TSKgel Amide-80 HR column (4.6 × 250 mm, φ = 5 μm) (Tosoh, Tokyo, Japan) at 65 °C, which was equilibrated with 85% solvent A (acetonitrile)/15% solvent B (100 mM ammonium formate buffer, pH 4.3). The elution was performed using a linear increase of solvent B (from 15 to 85%) over 90 min at a flow rate of 1 mL/min. Using a Waters 2475 Fluorescence Detector (Waters Corp., Milford, MA), the labelled sugars were detected at an excitation wavelength of 350 nm and an emission wavelength of 420 nm. The concentrations of mono- and oligosaccharides remaining in the spent medium were calculated based on the standard curves generated using similarly labelled standard sugars, and the data were normalized using the internal standard. The concentration of Fuc was measured separately with a colorimetric assay using fucose dehydrogenase (FDH) as described previously [74].

### Species abundances

From the DNA extracted with bead-beating and the phenol-chloroform method [41, 72], the relative abundance of each species within each sample was quantified using quantitative PCR (qPCR). qPCR was performed with a Thermal Cycler Dice Real-Time System (TaKaRa Bio., Kusatsu, Japan) as described previously [75]. The primer sets used in this study are listed in Supplementary Table 6. Primer specificity was confirmed by agarose gel electrophoresis following PCR using the genomic DNA of each species as templates (Extended Data Fig. 8). Known concentrations of genomic DNA extracted from each species were used as reference curves for species-specific quantification.

### Metagenome data mining and taxonomic profiling

Faecal metagenomic data were obtained from SRA (Sequence Read Archive; Accession Number: PRJEB6456) [36]. Data for the 98 infant-mother pairs were available for four different time points: the mother at the time of infant birth, and the infant at 0, 4, and 12 months of age. Of the 98 subjects, infants who were at least partially breastfed (exclusively breastfed or mixed-fed) at 4 months of age were chosen for analysis. Raw reads containing the letter ‘N’ (base pair not identified) were discarded. Reads containing the bacteriophage phiX DNA sequence were identified using Bowtie2 (version 2.3.4.1) [76] with pre-set options and the mapped reads were discarded. Removing of adapters, short reads (<50 bp), as well 3’-end quality trimming (score < 17), was performed using cutadapt (version 2.9) [77]. Next, reads that mapped against the human genome (GRCh 38) using Bowtie2 were also discarded.

The high-quality reads were used for taxonomic classification using Kraken2 version 2.1.2 [78] with standard database, and the abundance of each species was estimated using Bracken version 2.6.1 [79]. We also used METAnnotatorX2 v2.1 [80] with multiple manually curated RefSeq NCBI databases (http://probiogenomics.unipr.it/sw/METAnnotatorX2/METAnnotatorX2_BLASTn_taxonomy_databases.zip) to validate our results (Extended Data Fig. 9, Supplementary Table 7). The predicted taxonomy profiles were normalized based on the microorganisms’ genome sizes. Note that analyses were performed at the species level and did not differentiate between subspecies. Therefore, *B. longum* and *B. infantis* populations were both categorized within the *B. longum* group. If a read was aligned to more than one taxonomic sequence in the database with equal alignment scores, those taxonomies or genes were given a value of 1 divided by the number of hits. Taxonomic ratios were then calculated.

### Statistical analysis and quantification of priority effects

Statistical analysis was performed using R ver. 4.0.2 (www.r-project.org). The effect of assembly order on in vitro community composition was analysed with several multivariate techniques. To determine the effect size and significance of each covariate with permutational multivariate analysis of variance (PERMANOVA), the ‘envfit’ function in the package ‘vegan’ [81] was used, and ordination was performed using nonmetric multidimensional scaling (NMDS). The strength of priority effects for pairwise cultures was calculated using the equation proposed by Vannette and Fukami [35]. For four-species assembly, the strength of priority effects was assessed using regression analysis by relating the final population abundance of each species to its arrival order. Negative relationships between population abundance and arrival order were considered to be indicative of inhibitory priority effects [37]. The metagenomic data extracted from public databases, as described above, were also analysed with the ‘envfit’ function and visualized with NMDS as described above. We assessed whether the presence of a species at birth is associated with its dominance (>50% of the bifidobacterial community) at 4 months using Fisher’s exact test.

## Supplementary information

Extended Data Figures 1-9

Supplementary Tables 1-7

## Data Availability

The genome sequence of *B. bifidum* JCM 1254 was submitted to GenBank (accession numbers: BQJY01000001-BQJY01000062). Source data are provided with this paper. *B. longum* MCC10007 is available upon reasonable request.
