# Supplementary material for: Priority effects shape the structure of infant-type Bifidobacterium communities on human milk oligosaccharides
Source: ISME J. 2022 Jun 29;16(9):2265–79. doi: 10.1038/s41396-022-01270-3 (PMC9381805; doi:10.1038/s41396-022-01270-3)
Supplement: Supplementary file 1 — Extended Data Figures 1-9 [file 41396_2022_1270_MOESM1_ESM.pdf]

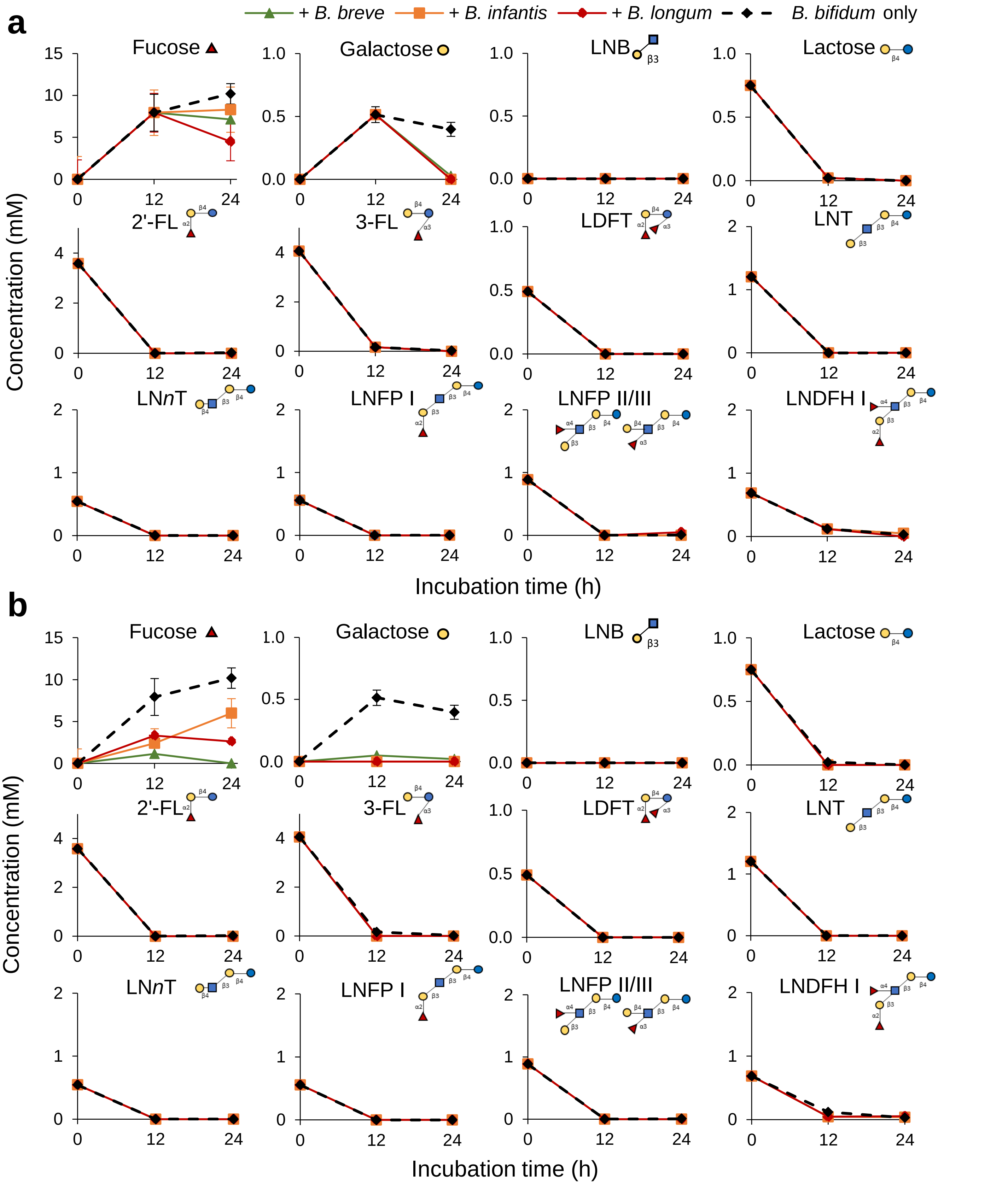

Extended Data Figure 1 | HMO consumption profiles of pairwise cultures with *B. bifidum* as the focal species. Culture supernatant was collected at each time point. The remaining sugars in the medium were labeled with 2-AA and analyzed by HPLC (as described in the Materials and Methods section). HMO consumption profiles are shown for pairwise cultures in which **a,** *B. bifidum* is inoculated first and the second species is inoculated 12 hours later, and **b,** *B. bifidum* is simultaneously cultured with other species. Black dotted lines indicate *B. bifidum* monoculture data (taken from Figure 1c), and competitors are indicated in different colors (green when cultured with *B. breve*, orange when cultured with *B. infantis*, and red when cultured with *B. longum*). Note that the presence of Glc/GlcNAc was not observed at the indicated time points. Data represent averages of biological quadruplicates, and error bars represent ± standard error.

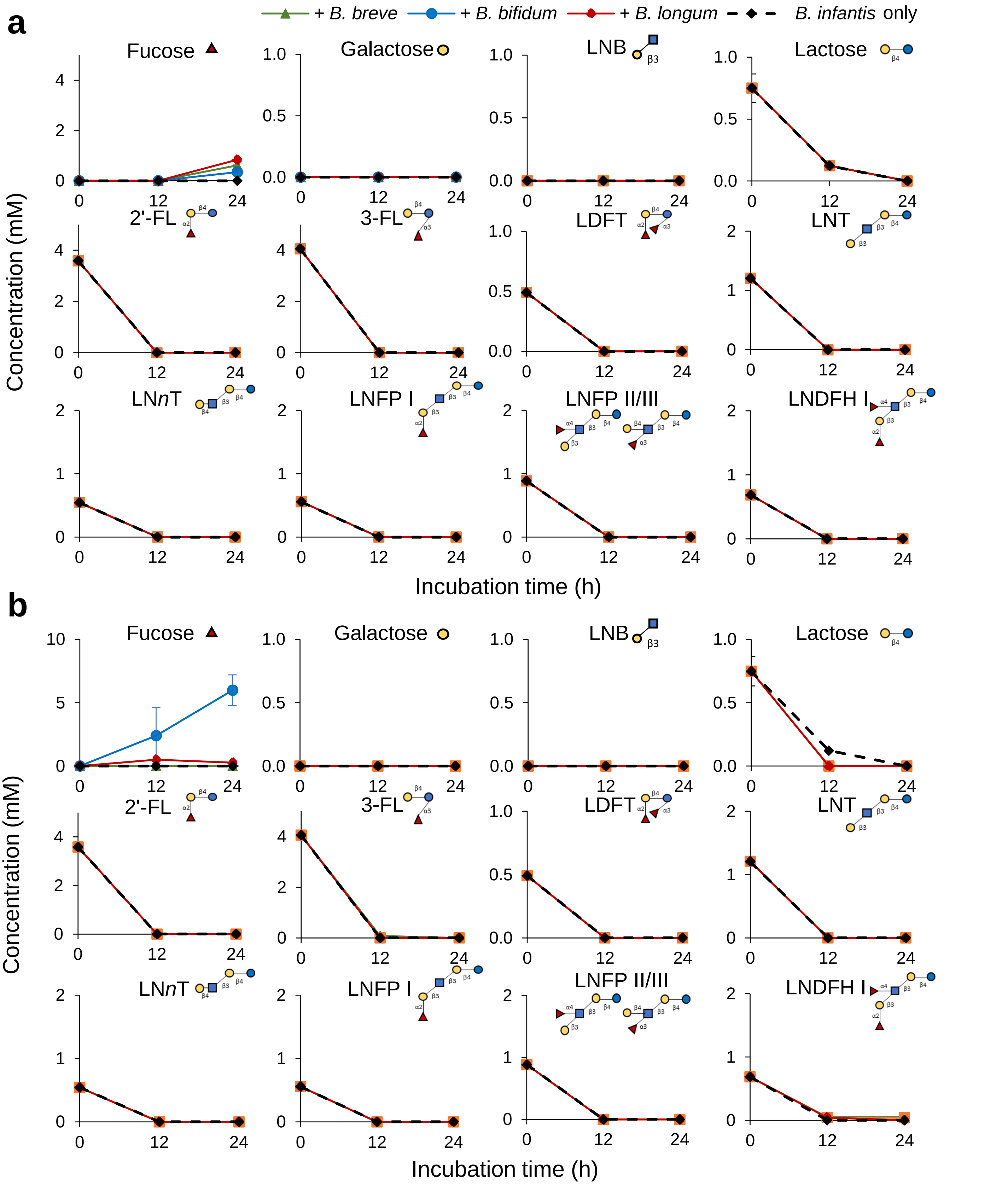

Extended Data Figure 2 | HMO consumption profiles of pairwise cultures with *B. infantis* as the focal species. Culture supernatant was collected at each time point. The remaining sugars in the medium were labeled with 2-AA and analyzed by HPLC (as described in the Materials and Methods section). HMO consumption profiles are shown for pairwise cultures in which **a,** *B. infantis* is inoculated first and the second species is inoculated 12 hours later, and **b,** *B. infantis* is simultaneously cultured with other species. Black dotted lines indicate *B. infantis* monoculture data (taken from Figure 1c), and competitors are indicated in different colors (green when cultured with *B. breve,* blue when cultured with *B. bifidum,* and red when cultured with *B. longum*). Note that the presence of Glc/GlcNAc was not observed at the indicated time points. Data represent averages of biological quadruplicates, and error bars represent ± standard error.

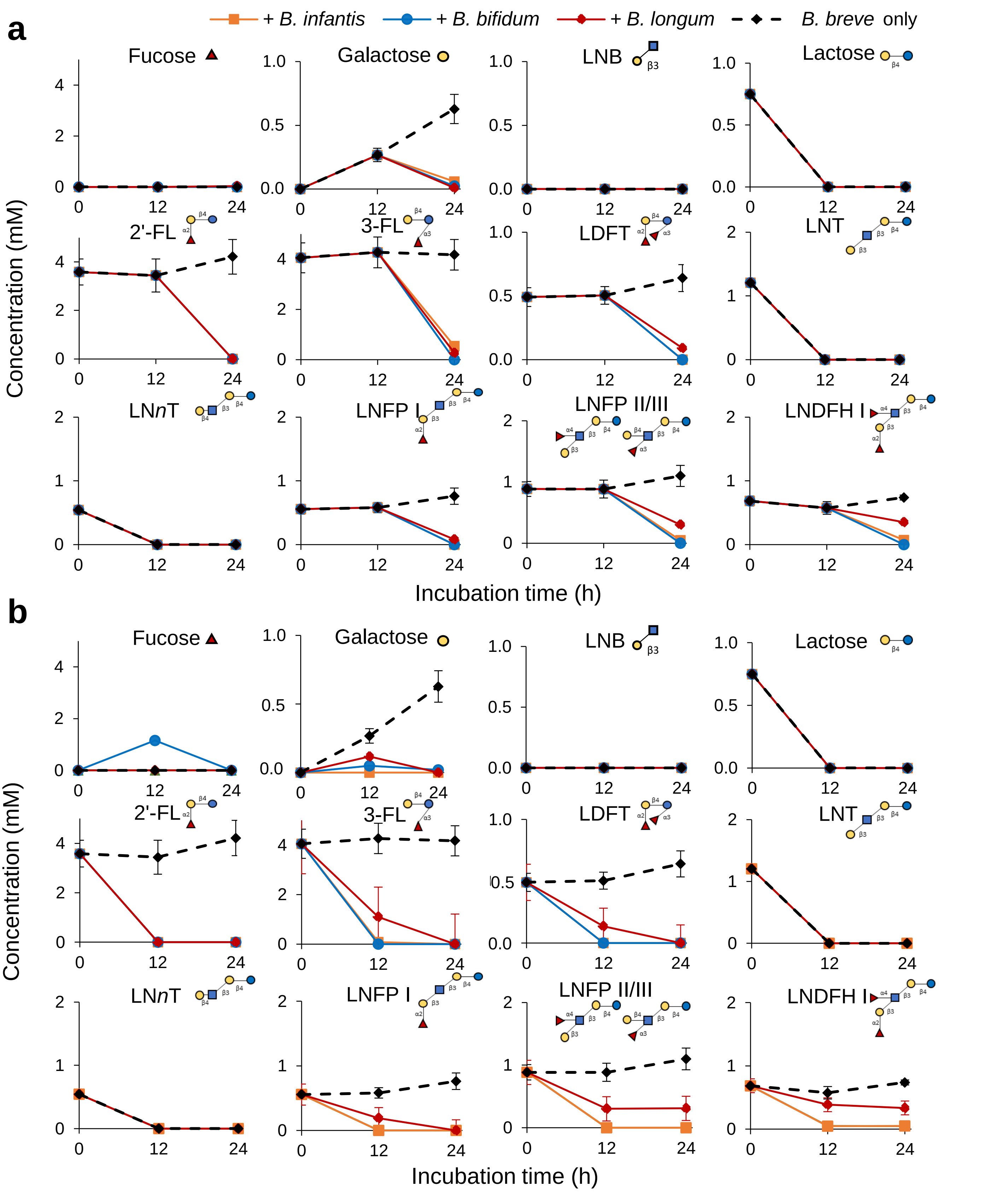

Extended Data Figure 3 | HMO consumption profiles of pairwise cultures with *B. breve* as the focal species. Culture supernatant was collected at each time point. The remaining sugars in the medium were labeled with 2-AA and analyzed by HPLC (as described in the Materials and Methods section). HMO consumption profiles are shown for pairwise cultures in which **a,** *B. breve* is inoculated first and the second species is inoculated 12 hours later, and **b,** *B. breve* is simultaneously cultured with other species. Black dotted lines indicate *B. breve* monoculture data (taken from Figure 1c), and competitors are indicated in different colors (orange when cultured with *B. infantis*, blue when cultured with *B. bifidum*, and red when cultured with *B. longum*). Note that the presence of Glc/GlcNAc was not observed at the indicated time points. Data represent averages of biological quadruplicates, and error bars represent ± standard error.

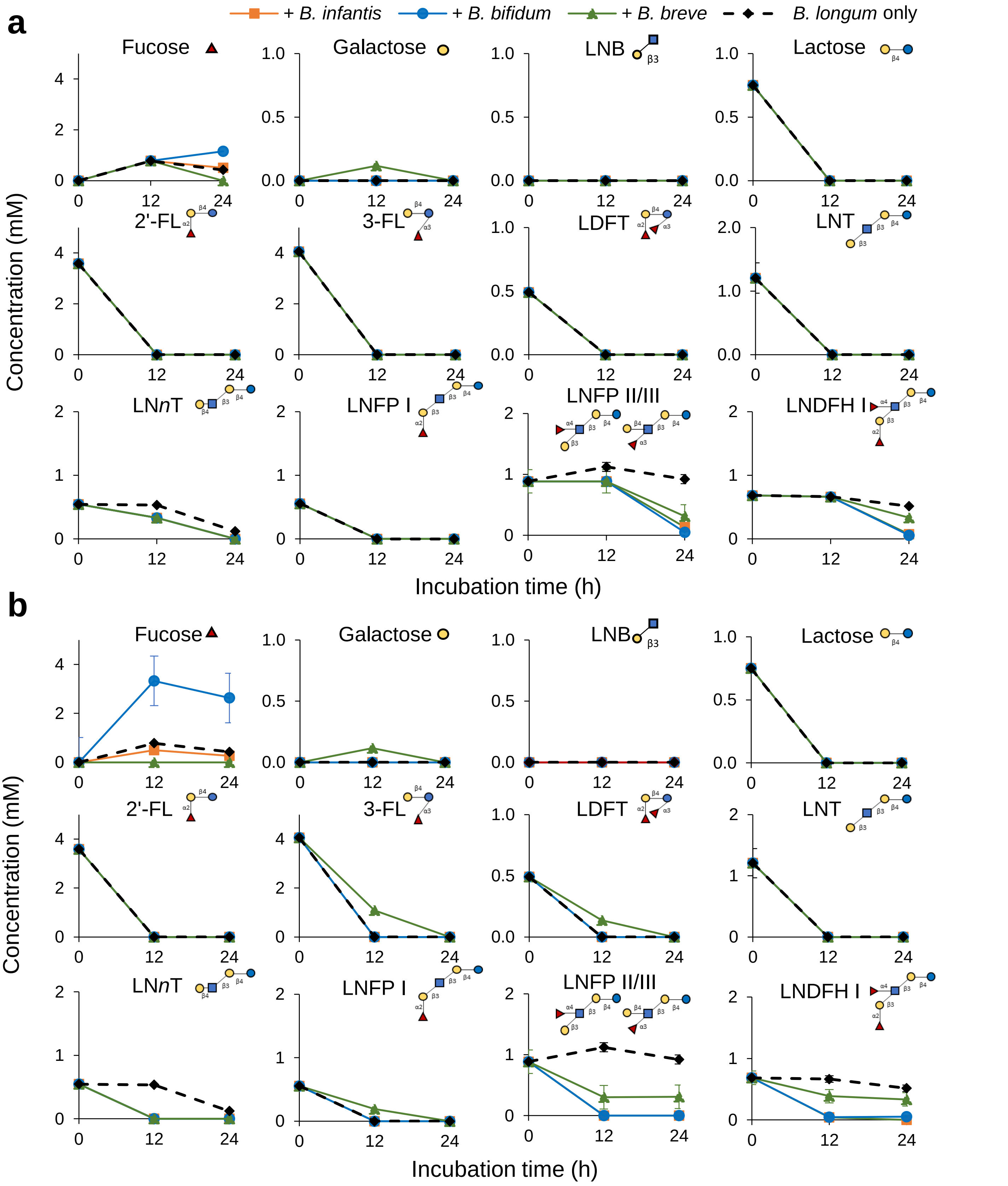

Extended Data Figure 4 | HMO consumption profiles of pairwise cultures with *B. longum* as the focal species. Culture supernatant was collected at each time point. The remaining sugars in the medium were labeled with 2-AA and analyzed by HPLC (as described in the Materials and Methods section). HMO consumption profiles are shown for pairwise cultures in which **a,** *B. longum* is inoculated first and the second species is inoculated 12 hours later, and **b,** *B. longum* is simultaneously cultured with other species. Black dotted lines indicate *B. breve* monoculture data (taken from Figure 1c), and competitors are indicated in different colors (orange when cultured with *B. infantis*, blue when cultured with *B. bifidum*, and green when cultured with *B. breve*). Note that the presence of Glc/GlcNAc was not observed at the indicated time points. Data represent averages of biological quadruplicates, and error bars represent ± standard error.

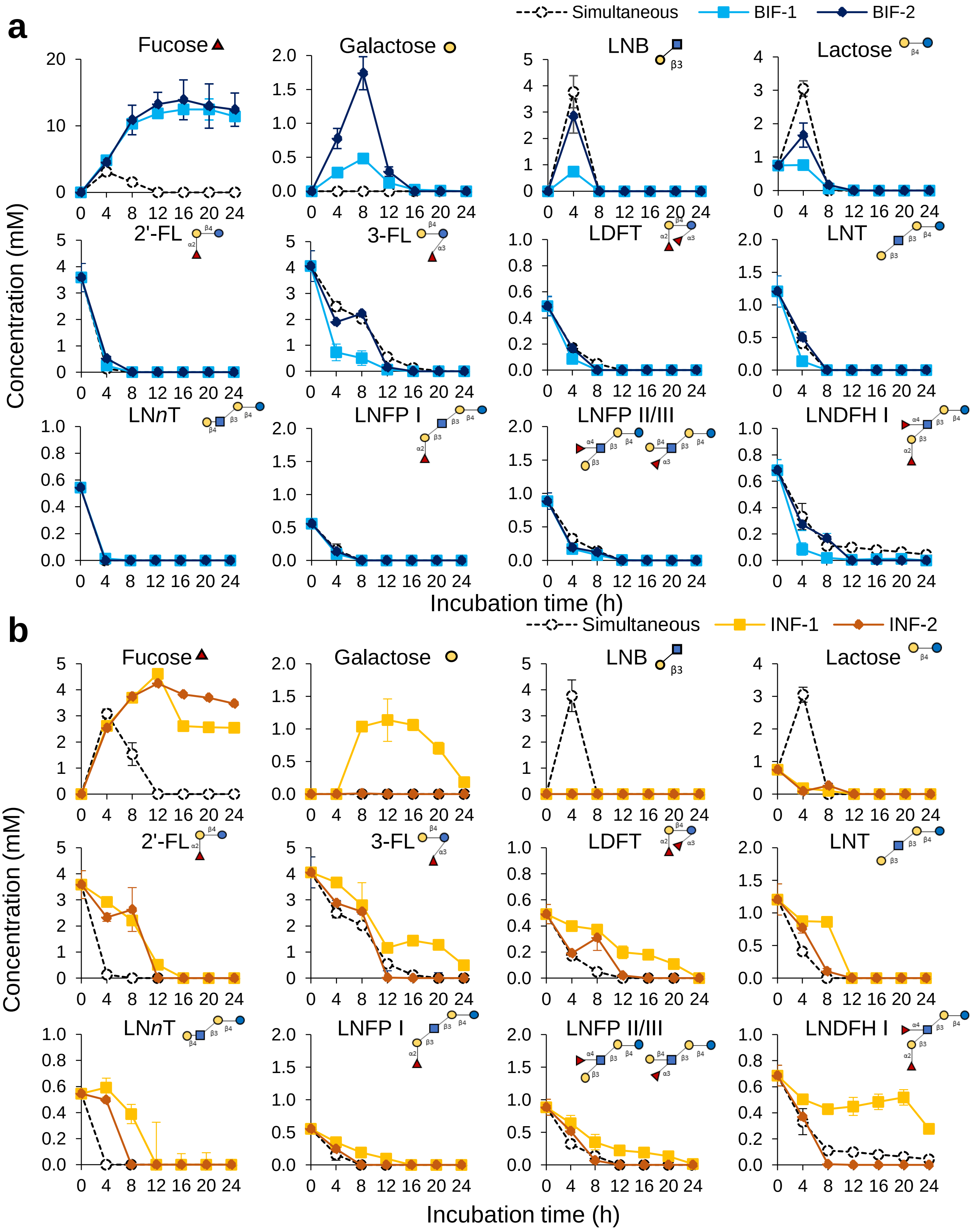

a
Simultaneous
BIF-1
BIF-2
Fucose
Galactose
LNB
Lactose
2'-FL
3-FL
LDFT
LNT
LNnT
LNFP I
LNFP II/III
LNDFH I
Concentration (mM)
Incubation time (h)
b
Simultaneous
INF-1
INF-2
Fucose
Galactose
LNB
Lactose
2'-FL
3-FL
LDFT
LNT
LNnT
LNFP I
LNFP II/III
LNDFH I
Concentration (mM)
Incubation time (h)

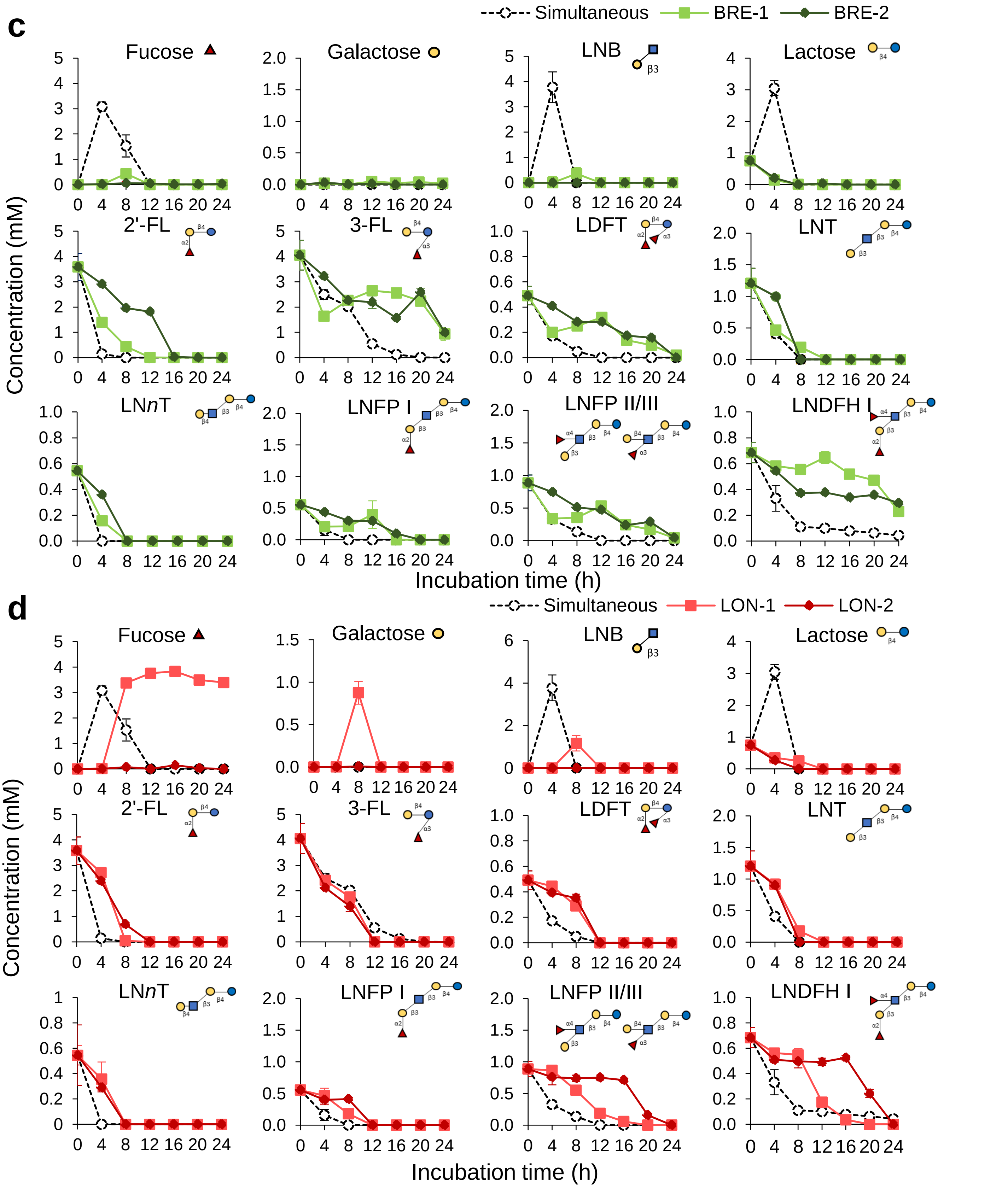

Extended Data Figure 5 | HMO consumption profiles for four-species assemblages. Culture supernatant was collected at each time point. The remaining sugars in the medium were labeled with 2-AA and analyzed by HPLC (as described in the Materials and Methods section). HMO consumption profiles are shown for four-species assemblages in which **a,** *B. bifidum* is inoculated first (light blue: BIF-1, dark blue: BIF-2), **b,** *B. infantis* is inoculated first (yellow: INF-1, orange: INF-2), **c,** *B. breve* is inoculated first (light green: BRE-1, dark green: BRE-2), **d,** *B. longum* is inoculated first (pink: LON-1, red: LON-2). The HMO consumption profile for the simultaneous culture is shown as the black dotted line (**a-d**). Note that the presence of Glc/GlcNAc was not observed at the indicated time points. Data represent averages of biological quadruplicates, and error bars represent ± standard error.

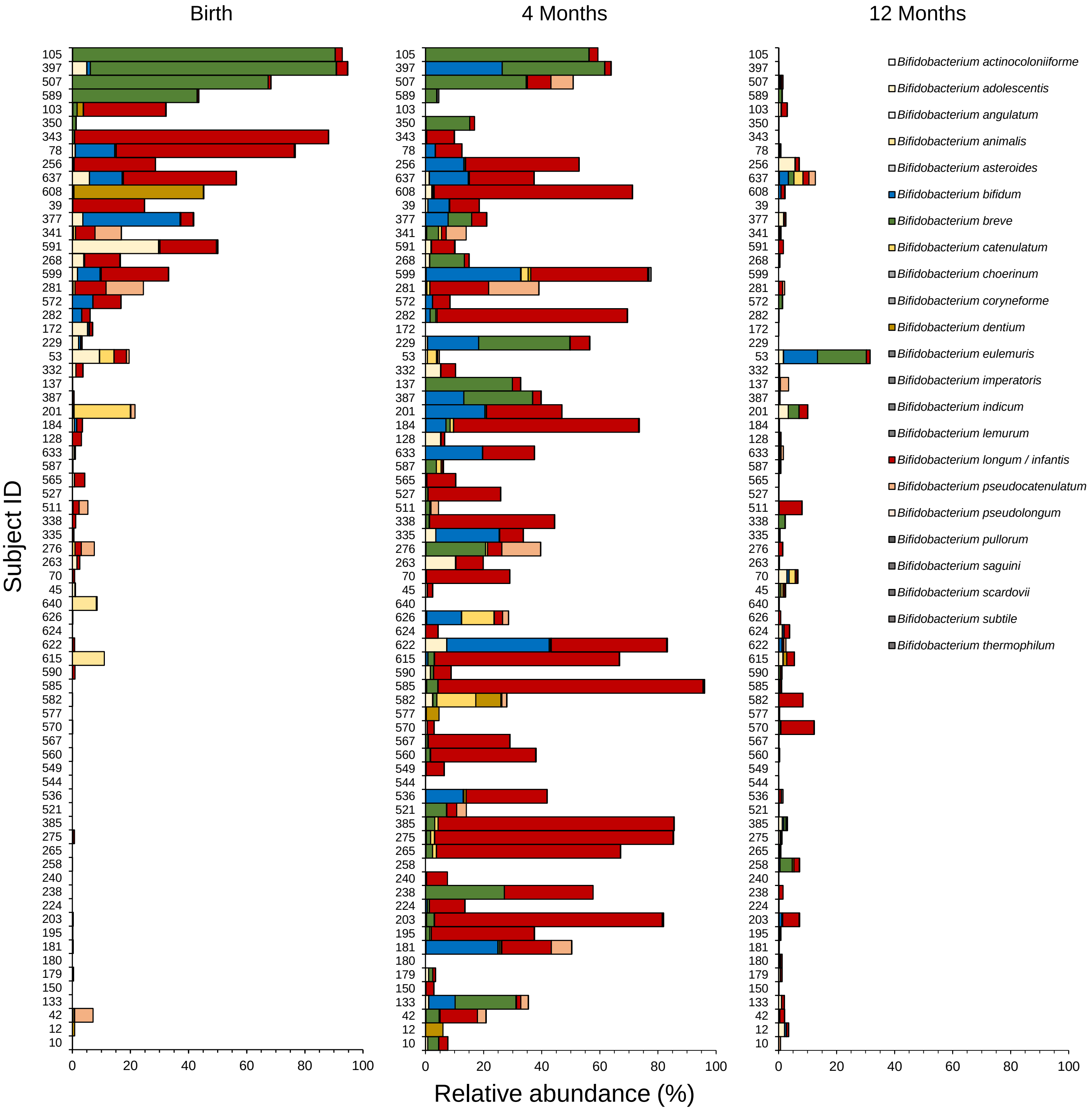

Extended Data Figure 6 | Relative abundance of each bifidobacterial species in the infant gut. The relative abundances of bifidobacterial species were extracted from the dataset published by Bäckhed et al. (2015) under the SRA Accession No. PRJEB6456. Relative abundances of bifidobacterial species at birth (left panel), 4 months of age (middle panel), and 12 months of age (right panel). Each bar represents one individual, which is ordered by *B. breve* abundance at birth. *B. breve* is indicated in green, *B. bifidum* in blue, and *B. longum* in red. Note that the analysis did not differentiate between the *B. longum* subspecies.

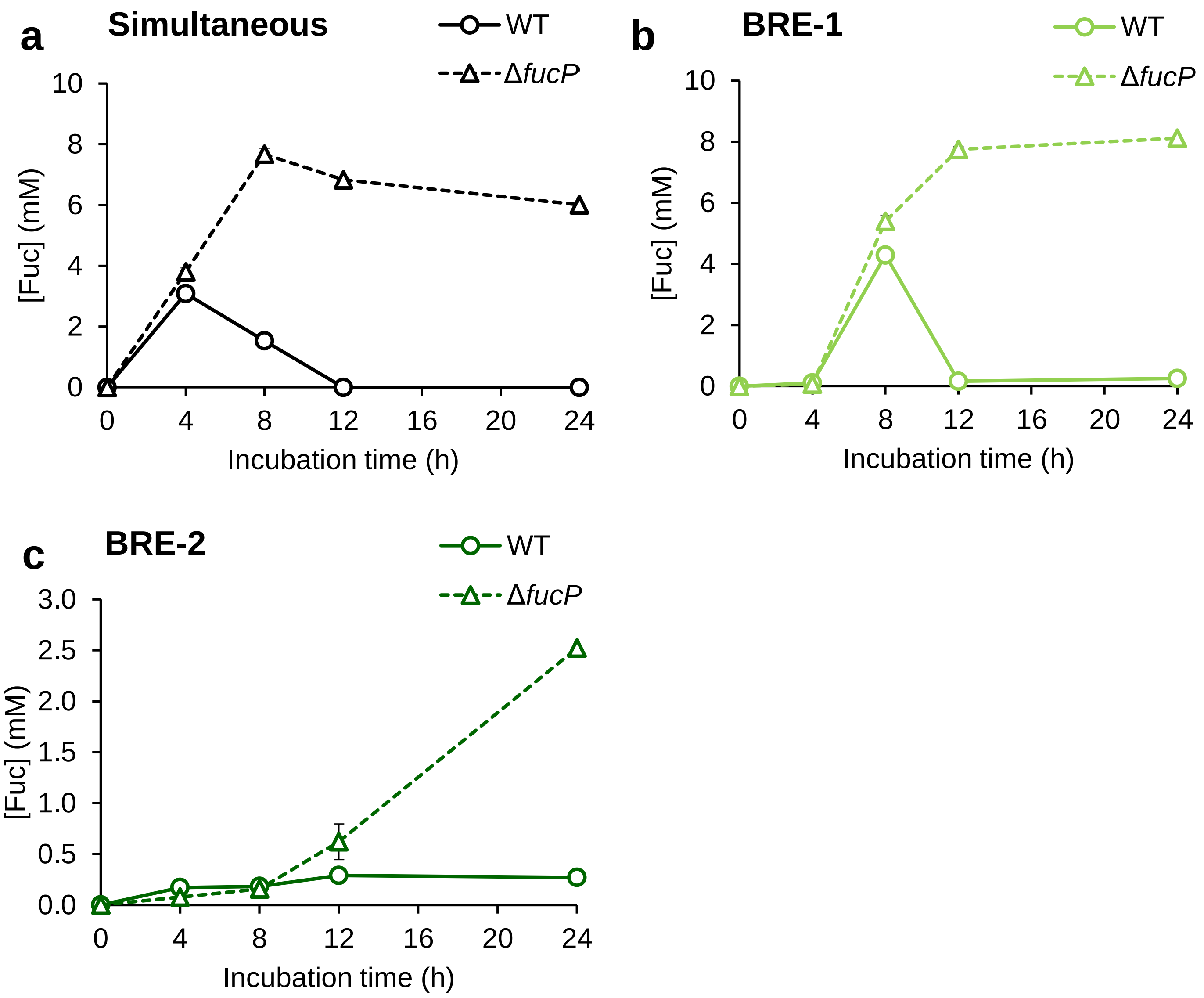

Extended Data Figure 7 | Concentration of remaining fucose in culturing medium in experiments with *B. breve* Δ*fucP* mutants. Culture supernatant was collected at each time point. The concentration of fucose remaining in the medium was measured using an fucose dehydrogenase assay, as described in the Materials and Methods Section. **a,** Simultaneous sequence. **b,** BRE-1 sequence. **c,** BRE-2 sequence. Solid lines indicate wild type (WT) *B. breve*, and dotted lines indicate Δ*fucP B. breve*. Data represent averages of biological triplicates, and error bars represent ± standard error.

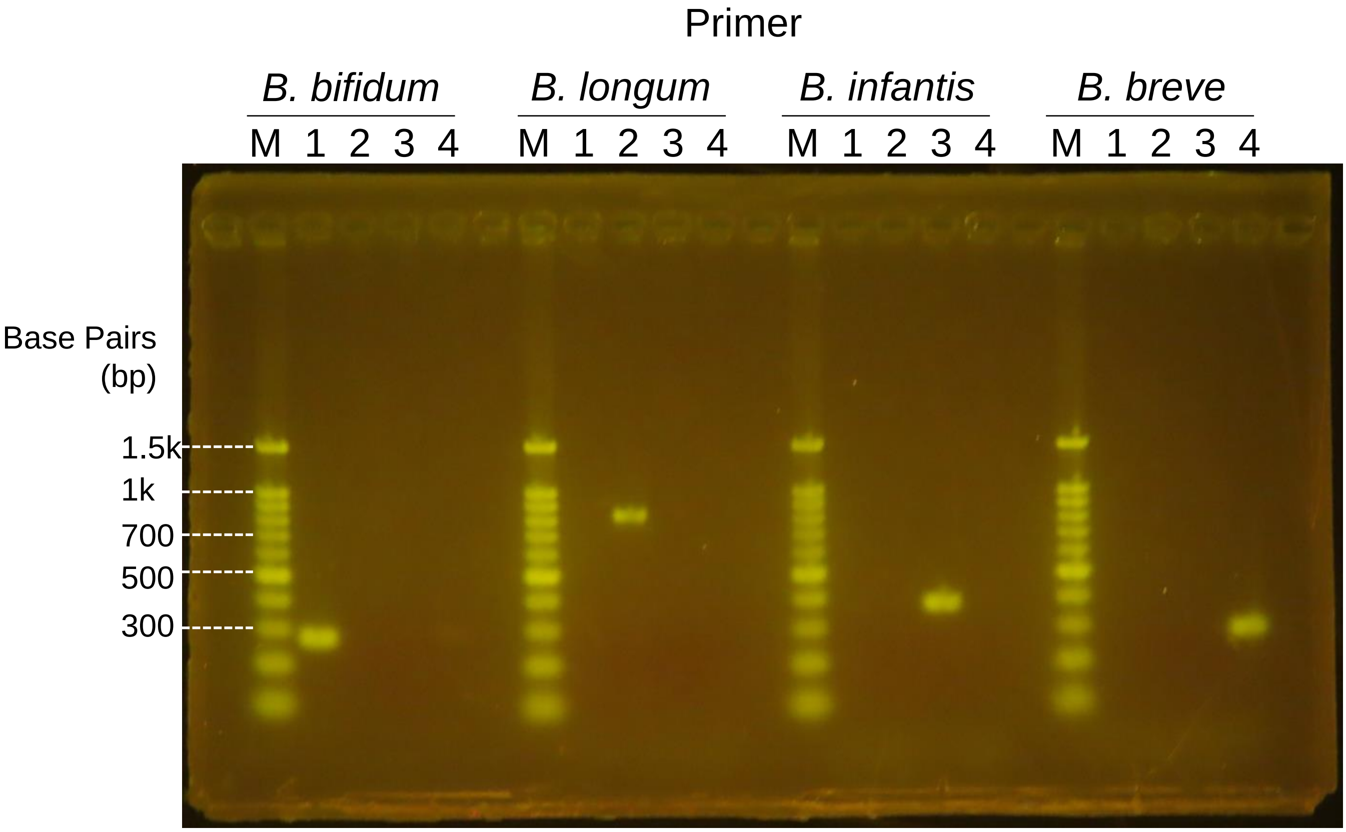

Extended Data Figure 8 | Gel electrophoresis to confirm primer specificity. A 30-cycle PCR was run with all primer pairs (see Supplementary Table 6 for primer sequences) using genomic DNA extracted from each of the species, and PCR products were subjected to an agarose gel electrophoresis to confirm that each primer only amplified the target species. Lanes are grouped by primer, and numbers indicate the template used for PCR. Lane M: 100 bp DNA ladder. Lane 1: Genomic DNA of *B. bifidum*. Lane 2: Genomic DNA of *B. longum*. Lane 3: Genomic DNA of *B. infantis*. Lane 4: Genomic DNA of *B. breve*.

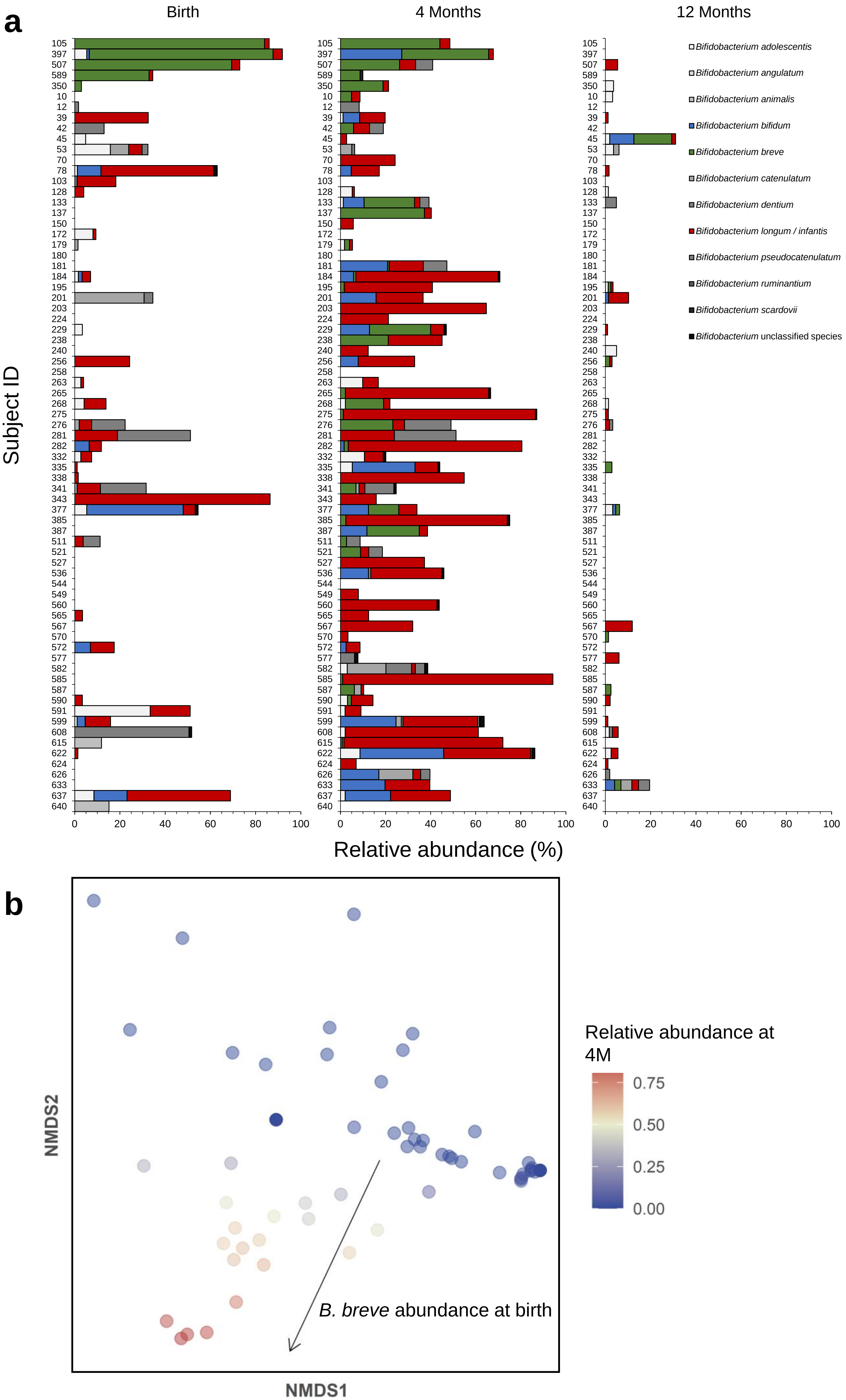
a
Birth
4 Months
12 Months
Subject ID
105 397 507 589 350 10 12 39 42 45 53 70 78 103 128 133 137 150 172 179 180 181 184 195 201 203 224 229 238 240 256 258 263 265 268 275 276 281 282 332 335 338 341 343 377 385 387 511 521 527 536 544 549 560 565 567 570 572 577 582 585 587 590 591 599 608 615 622 624 626 633 637 640
0 20 40 60 80 100
Relative abundance (%)
Bifidobacterium adolescentis
Bifidobacterium angulatum
Bifidobacterium animalis
Bifidobacterium bifidum
Bifidobacterium breve
Bifidobacterium catenulatum
Bifidobacterium dentium
Bifidobacterium longum / infantis
Bifidobacterium pseudocatenulatum
Bifidobacterium ruminantium
Bifidobacterium scardovii
Bifidobacterium unclassified species
b
NMDS2
NMDS1
B. breve abundance at birth
Relative abundance at 4M
0.75
0.50
0.25
0.00

Extended Data Figure 9 | Relative abundance of each bifidobacterial species in the infant gut, classified using METAnnotatorX2. a, The relative abundances of bifidobacterial species were extracted from the dataset published by Bäckhed et al. (2015) under the SRA Accession No. PRJEB6456. Relative abundances of bifidobacterial species, classified using METAnnotatorX2, at birth (left panel), 4 months of age (middle panel), and 12 months of age (right panel). Each bar represents one individual, which is ordered by *B. breve* abundance at birth. *B. breve* is indicated in green, *B. bifidum* in blue, and *B. longum* in red. Note that the analysis did not differentiate between the *B. longum* subspecies. Other *Bifidobacterium* species are indicated in varying shades of grey. **b,** Nonmetric multidimensional scaling (NMDS) ordination plots of bifidobacterial community data from the guts of infants who were at least partially breastfed (exclusively breastfed or mixed fed) at birth and at 4 months of age (n = 73) (see Supplementary Table 7). Each point corresponds to one individual. The color gradient indicates the *B. breve* abundance at 4 months of age. Statistically significant loadings are indicated as black arrows.
