# Supplementary material for: Priority effects shape the structure of infant-type Bifidobacterium communities on human milk oligosaccharides
Source: ISME J. 2022 Jun 29;16(9):2265–79. doi: 10.1038/s41396-022-01270-3 (PMC9381805; doi:10.1038/s41396-022-01270-3)
Supplement: Supplementary file 2 — Supplementary Tables 1-7 [file 41396_2022_1270_MOESM2_ESM.pdf]

Supplementary Tables

Supplementary Table 1 | Structures and initial concentrations of sugars (Lac and HMOs purified from pooled breastmilk) used in this study, at 1 % (*w/v*).

Oligosaccharide	Structure	Concentration (mM)
Lactose	Galβ1–4Glc	0.75 ± 0.12
2'-FL	Fucα1–2Galβ1–4Glc	3.58 ± 0.54
3-FL	Galβ1–4(Fucα1–3)Glc	4.06 ± 0.59
LDFT	Fucα1–2Galβ1–4(Fucα1–3)Glc	0.49 ± 0.07
LNT	Galβ1–3GlcNAcβ1–3Galβ1–4Glc	1.21 ± 0.24
LN*n*T	Galβ1–4GlcNAcβ1–3Galβ1–4Glc	0.54 ± 0.08
LNFP I	Fucα1–2Galβ1–3GlcNAcβ1–3Galβ1–4Glc	0.56 ± 0.08
LNFP II + III	Galβ1–3(Fucα1–4)GlcNAcβ1–3Galβ1–4Glc (LNFP II)	0.89 ± 0.12
	Galβ1–4(Fucα1–3)GlcNAcβ1–3Galβ1–4Glc (LNFP III)	
LNDFH I	Fucα1–2Galβ1–3(Fucα1–4)GlcNAcβ1–3Galβ1–4Glc	0.68 ± 0.08
Total		12.76 ± 1.52

* Abbreviations: 2'-FL, 2'-Fucosyllactose; 3-FL, 3-Fucosyllactose; LDFT, Lactodifucotetraose; LNT, Lacto-*N*-tetraose; LN*n*T, Lacto-*N*-*neo*tetraose; LNFP, Lacto-*N*-fucopentaose; LNDFH, Lacto-*N*-difucohexaose; Fuc, Fucose; Glc, Glucose; GlcNAc, *N*-Acetylglucosamine; Gal, Galactose

Supplementary Table 2 | Summary of genes related to HMO assimilation in the strains used in this study.

Name	Pathway	Annotation	Classification	*B. longum* subsp. *longum* MCC10007	*B. longum* subsp. *infantis* ATCC 15697	*B. breve* UCC2003	*B. bifidum* JCM 1254	Reference
GalK	Galactose catabolism	Galactokinase	EC 2.7.1.6	MCC10007_0432	Blon_2062	Bbr_0492	JCM1254_16870	(Inoue et al. 2011, Li et al. 2012)
GalE	Galactose catabolism	UDP-glucose 4-epimerase	EC 5.1.3.2	MCC10007_1574	Blon_0538	Bbr_0040	JCM1254_05330	(De Bruyn et al. 2013)
GalT	Galactose catabolism	UDP-glucose-hexose 1-phosphate uridylyl transferase	EC 2.7.7.12	MCC10007_0431	Blon_2063	Bbr_0491	JCM1254_16880	(Inoue et al. 2011)
Pgm	Galactose catabolism	Phosphoglucomutase	EC 5.4.2.2	MCC10007_1664	Blon_1766; Blon_2184	Bbr_0742;Bbr_1595	JCM1254_12450	NA
NagK	GlcNAc catabolism	Predicted *N*-acetyl-glucosamine kinase, ROK family	EC 2.7.1.59	MCC10007_1232	Blon_0879	Bbr_1250	JCM1254_01520	NA
NagA	GlcNAc catabolism	*N*-acetylglucosamine-6-phosphate deacetylase	EC 3.5.1.25	MCC10007_1229	Blon_0882	Bbr_1247	JCM1254_14260	NA
NagB	GlcNAc catabolism	Glucosamine-6-phosphate deaminase	EC 3.5.99.6	MCC10007_1230	Blon_0881	Bbr_1248	JCM1254_14270	NA
LnpA	LNB catabolism	1,3-β-galactosyl-*N*-acetylhexosamine phosphorylase	EC 2.4.1.211	MCC10007_1654	Blon_2174	Bbr_1587	JCM1254_01530, JCM1254_11570	(Kitaoka et al. 2005)
LnpB	LNB catabolism	*N*-acetylhexosamine 1-kinase	EC 2.7.1.162	MCC10007_1653	Blon_2173	Bbr_1586	JCM1254_01500	(Nishimoto and Kitaoka 2007)
LnpC	LNB catabolism	UDP-glucose-hexose 1-phosphate uridylyl transferase	EC 2.7.7.12	MCC10007_1652	Blon_2172	Bbr_1884	JCM1254_01490	(Nishimoto and Kitaoka 2007)
LnpD	LNB catabolism	UDP-glucose 4-epimerase	EC 5.1.3.2	MCC10007_1651	Blon_2171	Bbr_1585	JCM1254_01480	(Nishimoto and Kitaoka 2007)
FumB	Fucose catabolism	L-fucose mutarotase	EC 5.1.3.29	NA	Blon_2305; Blon_2337	NA	NA	(James et al. 2019)
FumC	Fucose catabolism	L-fuco-β-pyranose dehydrogenase	EC 1.1.1.122	MCC10007_0307	Blon_2308; Blon_2339	Bbr_1291; Bbr_1743	NA	(James et al. 2019)
FumD	Fucose catabolism	L-fuconolactone hydrolase	EC 3.1.1.-	MCC10007_0306	Blon_2306	Bbr_1290; Bbr_1741	NA	(James et al. 2019)
FumE	Fucose catabolism	L-fuconate dehydratase	EC 4.2.1.68	MCC10007_0308	Blon_0344;Blon_2309;Blon_2340	Bbr_1292; Bbr_1744	NA	(James et al. 2019)
FumF	Fucose catabolism	2-keto-3-deoxy-L-fuconate aldolase	EC 4.1.2.18	MCC10007_0305	Blon_2338	Bbr_1289, Bbr_1740	NA	(James et al. 2019)
FumG	Fucose catabolism	Predicted lactaldehyde reductase	EC 1.1.1.77	MCC10007_1571	Blon_0540	Bbr_1505	JCM1254_03860	(James et al. 2019)
Bga42A(BbgII)	GH	β-1,3/4/6-galactosidase	GH42	MCC10007_0485	Blon_2016	Bbr_0529	JCM1254_05840	(Goulas et al. 2009, Viborg et al. 2014, Ambrogi et al. 2019)
Bga2A(BbgIV)	GH	β-1,4-galactosidase	GH2	MCC10007_0744	Blon_2334	Bbr_1552	JCM1254_05290	(Goulas et al. 2009, Yoshida et al. 2012, Ambrogi et al. 2019)
BbgIII	GH	Extracellular β-1,4-galactosidase	GH2	NA	NA	NA	JCM1254_06870	(Miwa et al. 2010)
Hex1	GH	β-1,3/4/6-*N*-acetylglucosaminidase	GH20	NA	Blon_0459	Bbr_1556	JCM1254_03400	(Garrido et al. 2012)
Hex1′	GH	β-1,3/4/6-*N*-acetylglucosaminidase	GH20	MCC10007_1361	Blon_0732	NA	NA	(Garrido et al. 2012)
Hex2	GH	β-1,3/4-*N*-acetylglucosaminidase	GH20	NA	Blon_2355	NA	NA	(Garrido et al. 2012)
BbhI	GH	Extracellular β-1,3-*N*-acetylglucosaminidase	GH20	NA	NA	NA	JCM1254_09260	(Miwa et al. 2010)
BiAfcA	GH	α-1,2-L-fucosidase	GH95	MCC10007_0304	Blon_2335	Bbr_1288	NA	(Sela et al. 2012)
BiAfcB	GH	α-1,3/4-L-fucosidase	GH29	NA	Blon_2336	NA	NA	(Sela et al. 2012)
AfcA	GH	Extracellular α-1,2-L-fucosidase	GH95	NA	NA	NA	JCM1254_17170	(Katayama et al. 2004)
AfcB	GH	Extracellular α-1,3/4-L-fucosidase	GH29	NA	NA	NA	JCM1254_05790	(Ashida et al. 2009)
LnbB	GH	Extracellular lacto-*N*-biosidase	GH20	NA	NA	NA	JCM1254_04020	(Wada et al. 2008)
LnbX	GH	Extracellular lacto-*N*-biosidase	GH136	MCC10007_1471	NA	NA	NA	(Sakurama et al. 2013)
GlcP(GalP)	Transport	Glucose; galactose transporter	MFS; SP Family	MCC10007_1663	NA	NA	NA	(Parche et al. 2006)
GlcU	Transport	Glucose transporter	DMT; GRP Family	NA	NA	NA	JCM1254_04340	(Briczinski et al. 2008; Briczinski et al. 2009)
PtsG	Transport	Glucose transporter	PTS; Glc Family	MCC10007_1662	Blon_2183	Bbr_1594	JCM1254_12500	(Parche et al. 2007)
Blon_1383	Transport	Predicted galactose transporter	MFS; SSS Family	NA	Blon_1383	NA	NA	NA
FucP	Transport	Fucose transporter	MFS; FHS Family	NA	Blon_2307	Bbr_1742	NA	(Egan et al. 2014, Higgins et al. 2021)
LacS	Transport	Lactose transporter	MFS; GPH Family	MCC10007_0745	Blon_2331;Blon_2332	Bbr_1551	JCM1254_05300	(O'Connell Motherway et al. 2013)
GltABC	Transport	LNB transporter; predicted LNT transporter	ABC; CUT1 Family	MCC10007_1655-MCC10007_1657	Blon_2175-2177	Bbr_1588-1590	JCM1254_01540-01560	(Suzuki et al. 2008, Garrido et al. 2011, Katoh et al. 2020)
Blon_0883-0885	Transport	LNB transporter*	ABC; CUT1 Family	NA	Blon_0883-0885	NA	NA	(Garrido et al. 2011)
Blon_2345-2347	Transport	LN*n*T transporter*	ABC; CUT1 Family	NA	Blon_2345-2347	NA	NA	(Garrido et al. 2011)
Blon_2342-2344	Transport	LN*n*T (low affinity) transporter*	ABC; CUT1 Family	NA	Blon_2342-2344	NA	NA	(Garrido et al. 2011)
Bbr_1554	Transport	LN*n*T transporter	ABC; CUT1 Family	NA	Blon_0462^^	Bbr_1554	NA	(James et al. 2016)
FL1	Transport	2′-FL; 3-FL transporter	ABC; CUT1 Family	NA	Blon_0341-0343	NA	NA	(Sakanaka et al. 2019)
FL2	Transport	2′-FL; 3-FL; LDFT; LNFPI transporter	ABC; CUT1 Family	MCC10007_0309-MCC10007_0311	Blon_2202-2204	NA	NA	(Sakanaka et al. 2019)
Blon_2350	Transport	Predicted HMO transporter*	ABC; CUT1 Family	NA	Blon_2350	NA	NA	(Garrido et al. 2011)
Blon_2351	Transport	Predicted HMO transporter*	ABC; CUT1 Family	NA	Blon_2351	NA	NA	(Garrido et al. 2011)
Blon_2352	Transport	Predicted HMO transporter*	ABC; CUT1 Family	NA	Blon_2352	NA	NA	(Garrido et al. 2011)
Blon_2354	Transport	Predicted HMO transporter*	ABC; CUT1 Family	NA	Blon_2354	NA	NA	(Garrido et al. 2011)

^^ ortholog is truncated

* Based on *in vitro* binding assays

Strains that possess the listed gene are highlighted in green

Supplementary Table 3 | Quantification of priority effects in pairwise cultures. Priority effects were quantified based on the equation proposed by Vannette and Fukami[30]. The strength of priority effects for species *A* when cultured with species B (P_{AB}) is calculated by the log of the ratio between the absolute abundance (number of gene copies per ng of DNA + 1) of species *A* when it was introduced after species *B*, $D(A)_{BA}$, and when it was introduced before species *B*, $D(A)_{AB}$. $P_{AB} = \ln[\frac{D(A)_{BA}}{D(A)_{AB}}]$.

		Co-Culture Species			
		B. bifidum	*B. breve*	*B. infantis*	*B. longum*
Focal Species	*B. bifidum*		-2.88	-6.80	-1.88
	B. breve	-4.94		-4.84	2.94
	B. infantis	-0.60	-3.60		-3.81
	B. longum	-5.08	0.32	-11.91	

Supplementary Table 4 | PERMANOVA of the covariant relationship between the concentration of sugars remaining in the culture medium at 24 h, and the final community structure *in vitro* (Significance levels: * $p < 0.05$, ** $p < 0.01$, *** $p < 0.001$).

Variables (Sugar remaining in the medium at 24 h)	NMDS1	NMDS2	R^2	p	
Fuc	0.94921	-0.31465	0.4349	4.00E-04	***
GlcNAc/Glc	0.97376	-0.22759	0.1672	0.050	
Gal	-0.90301	-0.42962	0.124	0.091	
LNB	0	0	0	1.000	
Lac	0	0	0	1.000	
2′-FL	0	0	0	1.000	
3-FL	-0.90271	-0.43025	0.3518	0.002	**
LDFT	-0.90271	-0.43026	0.0989	0.132	
LNT	0	0	0	1.000	
LN*n*T	0	0	0	1.000	
LNFP I	0	0	0	1.000	
LNFP II/III	-0.90269	-0.43028	0.2096	0.021	*
LNDFH I	-0.9027	-0.43027	0.4453	2.00E-04	***

Supplementary Table 5 | PERMANOVA of the covariant relationship between the bifidobacterial community structure at 4 months of age, and the abundances of each bifidobacterial species at the time of birth, based on *in vivo* data from Bäckhed et al. (2015), with taxonomic classifications performed using Kraken2 and Bracken (Significance levels: * p < 0.05, ** p < 0.01, *** p < 0.001).

Variables (*Bifidobacterium* abundances at birth)	NMDS1	NMDS2	R^2	p	
Bifidobacterium actinocoloniiforme	0.000	0.000	0.000	1	
Bifidobacterium adolescentis	0.787	-0.617	0.016	0.432	
Bifidobacterium angulatum	0.994	-0.106	0.003	0.866	
Bifidobacterium animalis	-0.957	-0.290	0.092	0.052	.
Bifidobacterium asteroides	0.000	0.000	0.000	1.000	
Bifidobacterium bifidum	0.150	0.989	0.007	0.656	
Bifidobacterium breve	-0.984	0.177	0.092	0.0499	*
Bifidobacterium catenulatum	0.662	0.750	0.008	0.564	
Bifidobacterium choerinum	0.000	0.000	0.000	1.000	
Bifidobacterium coryneforme	0.000	0.000	0.000	1.000	
Bifidobacterium dentium	0.998	-0.057	0.005	0.607	
Bifidobacterium eulemuris	-0.990	-0.139	0.001	0.920	
Bifidobacterium imperatoris	0.553	0.833	0.007	0.666	
Bifidobacterium indicum	0.000	0.000	0.000	1.000	
Bifidobacterium lemurum	0.862	-0.508	0.002	0.820	
Bifidobacterium longum / *infantis*	0.706	0.708	0.015	0.496	
Bifidobacterium pseudocatenulatum	-0.064	-0.998	0.022	0.374	
Bifidobacterium pseudolongum	-0.980	0.200	0.011	0.418	
Bifidobacterium pullorum	0.995	-0.100	0.008	0.654	
Bifidobacterium saguini	0.026	1.000	0.001	0.927	
Bifidobacterium scardovii	0.902	-0.431	0.001	0.922	
Bifidobacterium subtile	-0.151	-0.989	0.010	0.553	
Bifidobacterium thermophilum	0.840	-0.543	0.004	0.756	

Supplementary Table 6 | List of primers used in this study

Species		Primer Sequence	Reference
Bifidobacterium bifidum	F	5′-CCACATGATCGCATGTGATTG -3′	(Matsuki et al. 1998)
	R	5′-CCGAAGGCTTGCTCCCAAA -3′	
Bifidobacterium breve	F	5′-CCGGATGCTCCATCACAC -3′	(Matsuki et al. 1998)
	R	5′-ACAAAGTGCCTTGCTCCCT -3′	
Bifidobacterium longum	F	5′- ACATCCAGGACCGTAACCTG -3′	(Toda et al. 2019)
subspecies *infantis*	R	5′- GCTTGTGCAGCTCCGTCT -3′	
Bifidobacterium longum	F	5′- TTCCAGTTGATCGCATGGTC -3′	(Matsuki et al. 1998)
subspecies *longum*	R	5′- GGGAAGCCGTATCTCTACGA -3′	

Supplementary Table 7 | PERMANOVA of the covariant relationship between the bifidobacterial community structure at 4 months of age, and the abundances of each bifidobacterial species at the time of birth, based on *in vivo* data from Bäckhed et al. (2015), with taxonomic classifications performed using METAnnotatorX2 (Significance levels: * $p < 0.05$, ** $p < 0.01$, *** $p < 0.001$).

Variables (*Bifidobacterium* abundances at birth)	NMDS1	NMDS2	R^2	p	
Bifidobacterium adolescentis	-0.35181	0.93607	0.0078	0.7615	
Bifidobacterium angulatum	0	0	0	1	
Bifidobacterium animalis	0.51601	0.85658	0.0019	0.957	
Bifidobacterium bifidum	0.73276	-0.68049	0.0087	0.7404	
Bifidobacterium breve	-0.42509	-0.90515	0.327	0.0001	***
Bifidobacterium catenulatum	-0.96156	-0.27459	0.0057	0.8295	
Bifidobacterium dentium	-0.21366	0.97691	0.0786	0.0593	
Bifidobacterium longum / *infantis*	0.34135	0.93994	0.0605	0.1062	
Bifidobacterium pseudocatenulatum	-0.91103	0.41233	0.0557	0.1165	
Bifidobacterium ruminantium	0	0	0	1	
Bifidobacterium scardovii	0	0	0	1	
Bifidobacterium unclassified species	0.8073	-0.59014	0.0072	0.777	

Supplementary References

1. Inoue K, Nishimoto M, Kitaoka M. One-pot enzymatic production of 2-acetamido-2-deoxy-D-galactose (GalNAc) from 2-acetamido-2-deoxy-D-glucose (GlcNAc). *Carbohydr Res* 2011; **346**: 2432–2436.

2. Li L, Liu Y, Wang W, Cheng J, Zhao W, Wang P. A highly efficient galactokinase from *Bifidobacterium infantis* with broad substrate specificity. *Carbohydr Res* 2012; **355**: 35–39.

3. De Bruyn F, Beauprez J, Maertens J, Soetaert W, De Mey M. Unraveling the leloir pathway of *Bifidobacterium bifidum*: Significance of the uridylyltransferases. *Appl Environ Microbiol* 2013; **79**: 7028–7035.

4. Kitaoka M, Tian J, Nishimoto M. Novel putative galactose operon involving lacto-*N*-biose phosphorylase in *Bifidobacterium longum*. *Appl Environ Microbiol* 2005; **71**: 3158–3162.

5. Nishimoto M, Kitaoka M. Practical preparation of lacto-*N*-biose I, a candidate for the bifidus factor in human milk. *Biosci Biotechnol Biochem* 2007; **71**: 2101–2104.

6. James K, Bottacini F, Contreras JIS, Vigoureux M, Egan M, Motherway MO, et al. Metabolism of the predominant human milk oligosaccharide fucosyllactose by an infant gut commensal. *Sci Rep* 2019; **9**: 1–20.

7. Goulas T, Goulas A, Tzortzis G, Gibson GR. Comparative analysis of four β-galactosidases from *Bifidobacterium bifidum* NCIMB41171: Purification and biochemical characterisation. *Appl Microbiol Biotechnol* 2009; **82**: 1079–1088.

8. Viborg AH, Katayama T, Abou Hachem M, Andersen MCF, Nishimoto M, Clausen MH, et al. Distinct substrate specificities of three glycoside hydrolase family 42 β-galactosidases from *Bifidobacterium longum* subsp. *infantis* ATCC 15697. *Glycobiology* 2014; **24**: 208–216.

9. Ambrogi V, Bottacini F, O'Sullivan J, O'Connell Motherway M, Linqiu C, Schoemaker B, et al. Characterization of GH2 and GH42 β-galactosidases derived from bifidobacterial infant isolates. *AMB Express* 2019; **9**.

10. Yoshida E, Sakurama H, Kiyohara M, Nakajima M, Kitaoka M, Ashida H, et al. *Bifidobacterium longum* subsp. *infantis* uses two different β-galactosidases for selectively degrading type-1 and type-2 human milk oligosaccharides. *Glycobiology* 2012; **22**: 361–368.

11. Miwa M, Horimoto T, Kiyohara M, Katayama T, Kitaoka M, Ashida H, et al. Cooperation of β-galactosidase and β-*N*-acetylhexosaminidase from bifidobacteria in assimilation of human milk oligosaccharides with type 2 structure. *Glycobiology* 2010; **20**: 1402–1409.

12. Garrido D, Ruiz-Moyano S, Mills DA. Release and utilization of *N*-acetyl-D-glucosamine from human milk oligosaccharides by *Bifidobacterium longum* subsp. *infantis*. *Anaerobe* 2012; **18**: 430–435.

13. Sela DA, Garrido D, Lerno L, Wu S, Tan K, Eom HJ, et al. *Bifidobacterium longum* subsp. *infantis* ATCC 15697 α-fucosidases are active on fucosylated human milk oligosaccharides. *Appl Environ Microbiol* 2012; **78**: 795–803.

14. Katayama T, Sakuma A, Kimura T, Makimura Y, Hiratake J, Sakata K, et al. Molecular cloning and characterization of *Bifidobacterium bifidum* 1,2-alpha-L-fucosidase (AfcA), a novel inverting glycosidase (glycoside hydrolase family 95). *J Bacteriol* 2004; **186**: 4885–93.

15. Ashida H, Miyake A, Kiyohara M, Wada J, Yoshida E, Kumagai H, et al. Two distinct α-L-

fucosidases from *Bifidobacterium bifidum* are essential for the utilization of fucosylated milk oligosaccharides and glycoconjugates. *Glycobiology* 2009; **19**: 1010–1017.

16. Wada J, Ando T, Kiyohara M, Ashida H, Kitaoka M, Yamaguchi M, et al. *Bifidobacterium bifidum* lacto-*N*-biosidase, a critical enzyme for the degradation of human milk oligosaccharides with a type 1 structure. *Appl Environ Microbiol* 2008; **74**: 3996–4004.

17. Sakurama H, Kiyohara M, Wada J, Honda Y, Yamaguchi M, Fukiya S, et al. Lacto-*N*-biosidase encoded by a novel gene of *Bifidobacterium longum* subspecies *longum* shows unique substrate specificity and requires a designated chaperone for its active expression. *J Biol Chem* 2013; **288**: 25194–25206.

18. Parche S, Beleut M, Rezzonico E, Jacobs D, Arigoni F, Titgemeyer F, et al. Lactose-over-glucose preference in *Bifidobacterium longum* NCC2705: glcP, encoding a glucose transporter, is subject to lactose repression. *J Bacteriol* 2006; **188**: 1260–1265.

19. Parche S, Amon J, Jankovic I, Rezzonico E, Beleut M, Barutcu H, et al. Sugar transport systems of Bifidobacterium longum NCC2705. *J Mol Microbiol Biotechnol* 2007; **12**: 9–19.

20. Briczinski EP, Loquasto JR, Barrangou R, Dudley EG, Roberts AM, Roberts RF. Strain-specific genotyping of *Bifidobacterium animalis* subsp. *lactis* by using single-nucleotide polymorphisms, insertions, and deletions. *Appl Environ Microbiol* 2009; **75**: 7501–7508.

21. Briczinski EP, Phillips AT, Roberts RF. Transport of glucose by *Bifidobacterium animalis* subsp. *lactis* occurs via facilitated diffusion. *Appl Environ Microbiol* 2008; **74**: 6941–6948.

22. Egan M, Motherway MO, Kilcoyne M, Kane M, Joshi L, Ventura M, et al. Cross-feeding by *Bifidobacterium breve* UCC2003 during co-cultivation with *Bifidobacterium bifidum* PRL2010 in a mucin-based medium. *BMC Microbiol* 2014; **14**: 1–14.

23. Higgins MA, Ryan KS. Generating a fucose permease deletion mutant in *Bifidobacterium longum* subspecies *infantis* ATCC 15697. *Anaerobe* 2021; **68**: 102320.

24. O'Connell Motherway M, Kinsella M, Fitzgerald GF, van Sinderen D. Transcriptional and functional characterization of genetic elements involved in galacto-oligosaccharide utilization by *Bifidobacterium breve* UCC2003. *Microb Biotechnol* 2013; **6**: 67–79.

25. Suzuki R, Wada J, Katayama T, Fushinobu S, Wakagi T, Shoun H, et al. Structural and thermodynamic analyses of solute-binding protein from *Bifidobacterium longum* specific for core 1 disaccharide and lacto-*N*-biose. *J Biol Chem* 2008; **283**: 13165–13173.

26. Garrido D, Kim JH, German JB, Raybould HE, Mills DA. Oligosaccharide binding proteins from *Bifidobacterium longum* subsp. *infantis* reveal a preference for host glycans. *PLoS One* 2011; **6:e17315**.

27. Katoh T, Ojima MN, Sakanaka M, Ashida H, Gotoh A, Katayama T. Enzymatic adaptation of *Bifidobacterium bifidum* to host glycans, viewed from glycoside hydrolyases and carbohydrate-binding modules. *Microorganisms* 2020; **8:481**.

28. James K, Motherway MOC, Bottacini F, van Sinderen D. *Bifidobacterium breve* UCC2003 metabolises the human milk oligosaccharides lacto-*N*-tetraose and lacto-*N*-*neo*-tetraose through overlapping, yet distinct pathways. *Sci Rep* 2016; **6**: 38560.

29. Sakanaka M, Hansen ME, Gotoh A, Katoh T, Yoshida K, Odamaki T, et al. Evolutionary adaptation in fucosyllactose uptake systems supports bifidobacteria-infant symbiosis. *Sci Adv* 2019; **5**: eaaw7696.
